# High-Altitude Living Shapes the Skin Microbiome in Humans and Pigs

**DOI:** 10.3389/fmicb.2017.01929

**Published:** 2017-10-06

**Authors:** Bo Zeng, Jiangchao Zhao, Wei Guo, Siyuan Zhang, Yutong Hua, Jingsi Tang, Fanli Kong, Xuewu Yang, Lizhi Fu, Kun Liao, Xianqiong Yu, Guohong Chen, Long Jin, Surong Shuai, Jiandong Yang, Xiaohui Si, Ruihong Ning, Sudhanshu Mishra, Ying Li

**Affiliations:** ^1^Farm Animal Genetic Resources Exploration and Innovation Key Laboratory of Sichuan Province, Sichuan Agricultural University, Chengdu, China; ^2^Division of Agriculture, Department of Animal Science, University of Arkansas, Fayetteville, AR, United States; ^3^Animal Husbandry and Technology Bureau of Daocheng County, Daocheng, China; ^4^Chongqing Academy of Animal Sciences, Chongqing, China; ^5^Pasturage Station of Tongjiang Agriculture Bureau, Bazhong, China

**Keywords:** skin microbiome, high altitude, Tibetans, Tibetan pigs, 16S rRNA

## Abstract

While the skin microbiome has been shown to play important roles in health and disease in several species, the effects of altitude on the skin microbiome and how high-altitude skin microbiomes may be associated with health and disease states remains largely unknown. Using 16S rRNA marker gene sequencing, we characterized the skin microbiomes of people from two racial groups (the Tibetans and the Hans) and of three local pig breeds (Tibetan pig, Rongchang pig, and Qingyu pig) at high and low altitudes. The skin microbial communities of low-altitude pigs and humans were distinct from those of high-altitude pigs and humans, with five bacterial taxa (*Arthrobacter, Paenibacillus, Carnobacterium*, and two unclassified genera in families *Cellulomonadaceae* and *Xanthomonadaceae*) consistently enriched in both pigs and humans at high altitude. Alpha diversity was also significantly lower in skin samples collected from individuals living at high altitude compared to individuals at low altitude. Several of the taxa unique to high-altitude humans and pigs are known extremophiles adapted to harsh environments such as those found at high altitude. Altogether our data reveal that altitude has a significant effect on the skin microbiome of pigs and humans.

## Introduction

Skin is the largest organ of the human body, and it serves as a physical barrier that protects the body from assault by pathogens and/or toxic materials. The skin is covered by a diverse array of microorganisms, most of which are harmless commensals. Different microbial communities are associated with different locations on the skin that are characterized as sebaceous, dry, and moist niches (Grice et al., 2009). Skin microbial diversity is also influenced by the environment, as well as host sex, species, and genetics (Fierer et al., 2008; Staudinger et al., 2011; Kueneman et al., 2014). Several studies have indicated a key role for the skin microbiome in the health and adaptability of different host species including mice (Grice et al., 2010), humans (Nakatsuji et al., 2013; Smeekens et al., 2014), dogs (Rodrigues Hoffmann et al., 2014), fish (Larsen et al., 2015), and amphibians (Walke et al., 2014). Notably, most of these studies have been performed at low altitude (Wolz et al., 2017); in contrast, little is known about the skin microbiome of humans and animals living at high altitudes (Wolz et al., 2017).

As the major interface between the mammalian body and the high-altitude environment, the skin of mammals living on the Tibetan Plateau is exposed to various extreme conditions such as hypobaric pressure, hypoxia, high ultraviolet radiation (UVR) and, throughout most of the year, a cold and dry environment (Beall, 2006; Jablonski and Chaplin, 2010). These conditions exert a substantial selective pressure on the skin microbiome. Studies conducted in low-altitude environments have revealed the important roles that the skin microbiome plays in health and disease by producing bacteriocins against skin pathogens (Bastos et al., 2009) and by boosting host immunity (Lai et al., 2010; Gallo and Nakatsuji, 2011). It is important to characterize the skin microbiome in mammals living at high altitude to determine whether the skin microbiome has adapted to the extreme environment and whether such adaptations aid in resistance to the development of skin diseases caused by extreme conditions such as UVR (Olsen et al., 2015). We hypothesize that the skin microbiomes of high-altitude mammals are different from those of low-altitude mammals, and that these microbiomes are specifically adapted to the high-altitude environment.

To test our hypothesis, we characterized the skin microbiomes of Chinese Han and Tibetans living on the Tibetan Plateau, the largest and highest plateau in the world (elevation 3750–3861 masl, meters above sea level), and of individuals living in the low-altitude Sichuan basin (elevation 319–1421 masl). Because porcine skin is strikingly similar to human skin with respect to general structure, thickness, hair follicle content, and pigmentation, and has shown to be an excellent biomedical model for human skin (Summerfield et al., 2015), we also examined the skin microbiomes of Tibetan pigs living at high and low altitude as well as low-altitude local pig breeds (Rongchang and Qingyu pigs).

## Materials and Methods

### Sampling, DNA Extraction, and Next-Generation Sequencing

**FIGURE 1:**
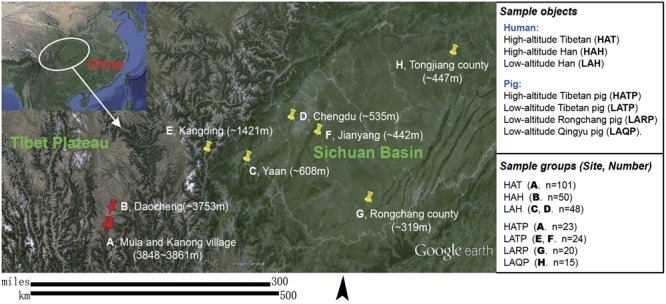
Subject groups and sampling locations. Three high-altitude and seven low-altitude sampling areas were labeled precisely on the Google Earth map using red and yellow indicator marks. **(A)** Two Tibetan villages in different river valleys of a mountainous area (separately marked on map) where both humans and pigs were sampled. **(B–D)** City area where urban Han residents were sampled. **(E,F)** Two pig-farms located in the suburbs of low-altitude cities; Tibetan pigs were transferred from the highland and raised at these farms for 6 months to 2 years. **(G)** A pig farm in the suburb of Rongchang county raising Rongchang pedigreed pigs. **(H)** Farmlands on hills near Tongjiang county where Qingyu pigs are raised in a fenced-in open area.

Two different human populations (the Hans and Tibetans, Supplementary Figure S1) living in the Daocheng area (high altitude, eastern edge of the Tibetan Plateau, 3750–3861 masl) and part of the Sichuan basin area (low altitude, 319–1421 masl; Hans only) in China (**Figure 1**) were recruited for this study. All human subjects were healthy and had no skin allergies or infections. Individuals who had antibiotic treatment or had applied any cosmetic cream or powder within 24 h were excluded. Relevant metadata including gender, age, and place of residence were recorded for each sample (Supplementary Table S1). Among the 101 Tibetan subjects included in this study, 22 lived in the same building as Tibetan pigs, with humans living on the second floor and pigs in the barn on the first floor. Tibetan pigs in both high and low altitude (as controls), together with two other low-altitude local pig breeds (Rongchang pig and Qingyu pig, **Figure 1**) were also included in this study. Humans (*n* = 199) were split into three groups based on race and altitude: the high-altitude Hans (HAH), low-altitude Hans (LAH), and high-altitude Tibetans (HAT). Pigs (*n* = 82) were similarly grouped: high-altitude Tibetan pigs (HATP), low-altitude Tibetan pigs (LATP), low-altitude Rongchang pigs (LARP), and low-altitude Qingyu pigs (LAQP). The geographical distributions of the individuals and pigs sampled in this study are shown in **Figure 1**. All experimental procedures were performed in accordance with the Institutional Review Board (IRB14044) and the Institutional Animal Care and Use Committee of the Sichuan Agricultural University under permit number DKY-B20140302. Written informed consent was obtained from each human participant in compliance with the Declaration of Helsinki.

A single skin sample was collected from humans (*n* = 199) and pigs (*n* = 82) using sterile swabs pre-wet with SCF-1 buffer [50 mM Tris buffer (pH 7.6), 1 mM EDTA (pH 8.0), and 0.5% Tween-20]. Swabs were rubbed vigorously over the area to be sampled (forehead for humans, back skin close to the neck for pigs) to collect surface material. The swab heads were then cut off and placed into 2 ml centrifuge tubes, snap-frozen in liquid nitrogen, and stored at -80°C until further study. The samples were collected between March 2013 and April 2014. The air temperature was recorded during sampling. To estimate the UVR intensity during the sampling period of time, we measured the UVR intensity at around 12:00–14:00 in several sunny and cloudy days, respectively, during September 2016 using an ultraviolet meter (UVAB/ST-513, spectrum range 280–400 nm, SENTRY, Taiwan). Average values ranged from 16.1 to 17.7 mW/cm^2^ in the high-altitude Daocheng district and from 7.8 to 9.0 mW/cm^2^ in the low-altitude Sichuan basin (Supplementary Figure S2).

Total bacterial DNA was extracted directly from the swab heads using a TIANamp Micro DNA Kit (TIANGEN Biotech, Beijing, China) according to the manufacturer’s instructions. DNA was quantified using a Qubit 3.0 Fluorometer (Life Technologies, Shanghai, China). 16S rRNA gene amplicons were produced and sequenced at the Beijing Genomics Institute (BGI Shenzhen, China). Briefly, variable region 4 of the 16S rRNA gene was amplified using the 515f/806r barcoded primer pair (515f: 5′-GTG CCA GCM GCC GCG GTA A-3′, 806r: 5′-XXX XXX GGA CTA CHV GGG TWT CTA AT-3′) (Caporaso et al., 2011). PCR (25 μL) contained 13 μL water, 10 μL PCR Master Mix, 0.5 μL each of the forward and reverse primers (10 μM), and 1.0 μL DNA template. Reaction was performed at 94°C for 3 min followed by 35 cycles of (94°C for 45 s, 50°C for 60 s, 72°C for 90 s). A final step at 72°C for 10 min was also included. Each sample was amplified in triplicate. PCR products (∼300 bp) were combined and purified, and then proceeded for library preparation by using MiSeq Reagent Kits v2 [Illumina China (Shanghai), Co., Ltd.]. High-throughput sequencing were conducted by using the Illumina MiSeq 2 × 250 protocol. Negative controls during DNA extraction and PCR were included to rule out any contamination from the swabs and kits.

### Sequence Processing and Analysis

Raw sequences were demultiplexed and denoised by using Usearch v9.0 with author recommended command lines (fastq_mergepairs and fastq_filter command). Clean, per-sample FASTA files were analyzed using QIIME v1.9.0 (Caporaso et al., 2010). Chimeric sequences were removed using UCHIME (Edgar et al., 2011), and operational taxonomic units (OTUs) were picked *de novo* using a 97% similarity threshold. Taxonomy assignment was performed by using uclust consensus taxonomy assigner against the Greengenes database (gg_13_5). The details of command lines and parameters used for sequence data analysis are presented in Supplementary Material.

We computed several alpha diversity metrics, including the Shannon index, the Simpson reciprocal, and the observed OTUs. To correct for differences in sequencing depth, we randomly subsampled the OTU table to a depth of 2820 sequences per sample 10 times before computing the alpha diversity metrics. To assess beta diversity, we computed jackknifed (10 sub-samplings at a depth of 3188 sequences per sample) unweighted and weighted UniFrac and Bray–Curtis distances, and visualized the matrices using principal coordinates analysis (PCoA). The phylogenetic tree was clustered using the Unweighted Pair Group Method with Arithmetic Mean (UPGMA), metadata coloring and legend generation was performed using EvolView (He et al., 2016).

### Statistical Analyses

A Mann–Whitney *U*-test was used to determine significant differences in alpha and beta diversity between groups. We used linear discriminant analysis (LDA) coupled with effect size (LEfSe) (Segata et al., 2011) to identify differentially abundant genera between groups and then created heat maps to visualize the mean relative abundances (per group) of the differentially abundant genera. Core microbiome in high altitude groups were picked out by using QIIME script. To determine the strength and significance of given factors in explaining microbiome variation between comparison groups, we performed a permutational multivariate analysis of variance (PERMANOVA) test. We also used SparCC to compute and generate the taxa-to-taxa correlation matrix data for significantly positively or negatively correlated taxa (Friedman and Alm, 2012), and network inference analyses were performed using Cytoscape (v3.3.0).

## Results

### High-Altitude Environment Is the Major Driver Shaping the Skin Microbiomes of Tibetans and Tibetan Pigs

In this study, 281 skin samples (199 human, 82 pig) were collected and processed for 16S rRNA gene amplicon sequencing and analysis. A total of 6,644,855 reads were produced by the Illumina MiSeq 2 × 250 sequencing run. After chimera checking and filtering out singleton OTUs, a total of 5,591,594 reads corresponding to 21,101 OTUs were retained in the dataset. On average, each sample had 532 OTUs and 19,899 reads (Supplementary Table S1).

**FIGURE 2:**
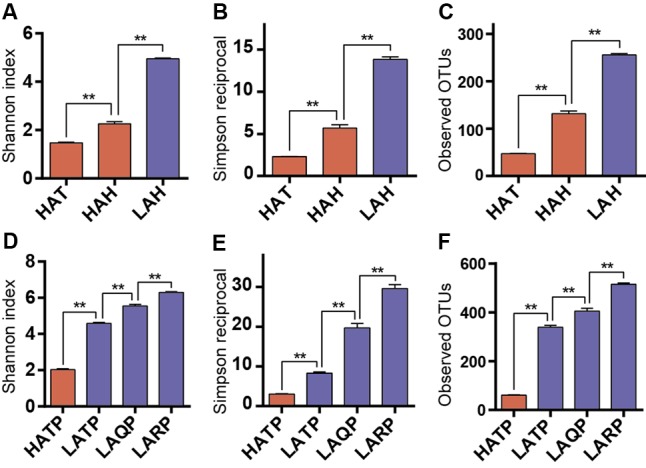
Alpha diversity differences between groups. Three metrics were used for comparison including Shannon index **(A,D)**, Simpson reciprocal **(B,E)**, and the number of observed OTUs **(C,F)** in both humans and pigs. Red and blue bars represent the high- and low-altitude skin groups, respectively. ^∗∗^*p* < 0.01, Mann–Whitney *U*-test.

To assess within-sample diversity, we calculated three alpha diversity metrics: the Shannon diversity index, the Simpson reciprocal, and the number of observed OTUs (**Figure 2**). Both the Hans and Tibetans at high altitude (HAH and HAT) had significantly less diverse skin microbiomes than Hans at low altitude (LAH; *p* < 0.01, **Figures 2A–C**). Interestingly, although both high-altitude human groups had lower skin community diversities than the low-altitude group, diversity also differed significantly between the HAH and HAT, with lower diversity microbial communities characterizing the HAT group (*p* < 0.01). Similarly, the diversity of the skin microbiome was significantly lower in HATP than in LATP, and also lower than low-altitude local breed pigs (Qingyu and Rongchang pigs, LAQP and LARP) (*p* < 0.01, **Figures 2D–F**). Additionally, although the skin microbial diversity was significantly higher in LATP compared to the HATP counterpart (*p* < 0.01), the skin microbial diversity of this group was still lower than that of the local breeds (*p* < 0.01).

**FIGURE 3:**
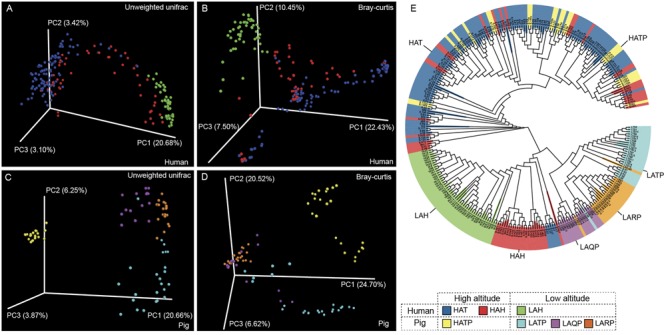
Jackknifed beta diversity analysis. **(A,C)** Unweighted UniFrac- and **(B,D)** Bray–Curtis-based principal coordinates analysis (PCoA) of human and pig skin microbiomes were performed based on unweighted UniFrac distances and Bray–Curtis distances to assess microbiome differences between high- and low-altitude groups and **(E)** UPGMA tree of all samples; clustering was based on unweighted UniFrac distances.

Between-group diversity was assessed using the unweighted and weighted UniFrac and Bray–Curtis distance metrics and visualized using PCoA (**Figure 3** and Supplementary Figure S3). Similarly to alpha diversity observations, the skin microbiome of the HAT group was significantly different from that of the LAH group (**Figures 3A,B**, *p* < 0.01, Supplementary Figure S4). The skin microbiome of the HAH group appeared to follow a gradient between the LAH and HAT groups, although the HAH and LAH groups differed significantly (*p* < 0.01, Supplementary Figure S4). Similarly, in pigs, the skin microbiome was significantly different between high- and low-altitude pigs (**Figures 3C,D**, *p* < 0.01, Supplementary Figure S4, HATP vs. LATP/LAQP/LARP). Interestingly, the skin microbial communities of high-altitude pigs were more similar to high-altitude humans than to low-altitude pigs (Supplementary Figure S3, HAT and HATP). The unweighted UniFrac distance between the HATP and HAT groups was significantly smaller than that between the HATP and LATP groups (Supplementary Figure S5, *p* < 0.01). Additionally, clusters on the phylogenetic tree (**Figure 3E**) are specific to altitude, with completely different clades associated with high and low altitude.

To determine whether altitude, age, gender, or a number of other demographic factors were significantly associated with skin microbiome diversity, we performed a PERMANOVA. Although most of these factors were significantly correlated to skin microbiome diversity (Supplementary Table S2, *p* < 0.01), the strongest association was observed with altitude in both humans and pigs (Supplementary Table S2, pseudo-*F* = 28.6 in humans and 22.4 in pigs).

### Identification of Bacterial Taxa Significantly Increased or Decreased in High- and Low-Altitude Humans and Pigs

We next examined bacterial community composition to identify taxa enriched in Tibetans and Tibetan pigs. At the phylum level, Proteobacteria (55.0%), Firmicutes (27.1%), and Actinobacteria (13.3%) were the three most abundant phyla in both humans and pigs, although inter-personal variation within each group was observed (Supplementary Figure S6). At the genus level, *Erwinia, Pseudomonas*, and *Acinetobacter* were the top 3 taxa in both human and pigs. The top 22 genera, comprising 72.38% of the total reads, are listed in Supplementary Figure S7.

**FIGURE 4:**
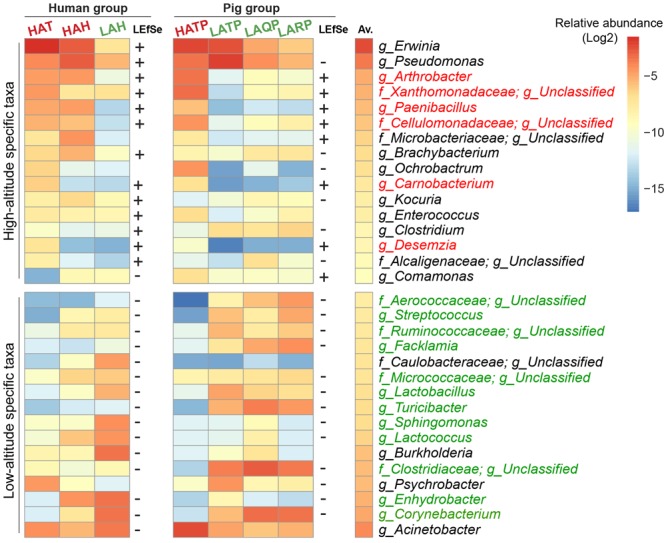
Skin bacterial taxa associated with high and low altitude. The average relative abundance of the top 16 LEfSe-identified bacterial taxa in humans and pigs is plotted as a heatmap. “+” indicates significantly high abundance in high-altitude groups, “–” significantly high abundance in low-altitude groups (*p* < 0.05, LDA cutoff = 2.0). Taxa that could not be annotated down to the family level or with conflicting results using LEfSe were omitted. Taxa names were colored (red, high altitude; green, low altitude) if LEfSe results were consistent in both human and pig groups. The Av. column indicates the total average relative abundance of each taxon across all samples.

To identify specific bacterial taxa significantly differentiating between the high- and low-altitude groups, we performed LEfSe analysis based on taxa present at a relative abundance of at least 0.1%. Among humans, 27 taxa were significantly increased in the high-altitude groups, with 45 taxa significantly decreased in the low-altitude groups. Among pigs, 11 taxa were significantly increased in the high-altitude groups, while 54 taxa were significantly increased in the low-altitude groups (*p* < 0.05, LDA cutoff = 2.0). Interestingly, six taxonomic groups (*Arthrobacter, Paenibacillus, Carnobacterium, Desemzia, Cellulomonadaceae*, and *Xanthomonadaceae*) were found more abundant in high-altitude samples and were detected on the skin of both Tibetan humans and pigs (**Figure 4**). *Erwinia* was the most abundant genus detected in humans at high altitude, and while also detected in pigs at high altitude, was not significantly higher in these pigs compared to their low-altitude counterparts. Additionally, 12 taxa (e.g., *Corynebacterium* spp., *Enhydrobacter* spp., *Lactococcus* spp., **Figure 4**) were consistently more abundant in both low-altitude humans and pigs compared to their high-altitude counterparts.

### Multiple Taxa Associated with High-Altitude Samples Are Strongly and Positively Correlated

**FIGURE 5:**
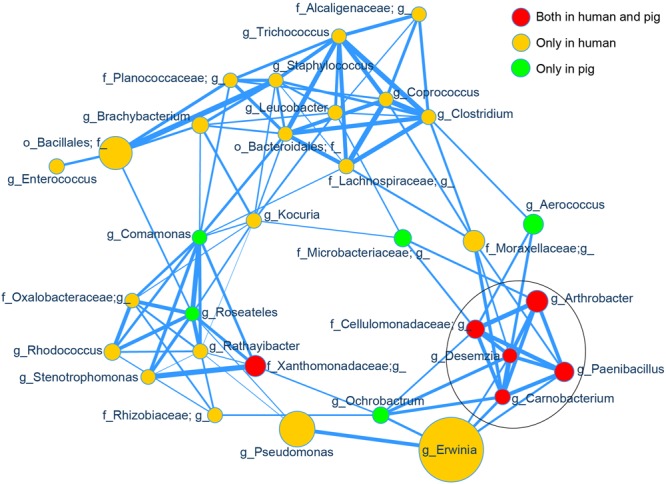
Correlation network of the high altitude specific skin taxa. The relative abundances of the top 32 high altitude specific skin bacterial genera identified by LEfSe were used as input for SparCC correlation analysis and visualized via a customized Cytoscape network. Data for all samples (both human and pig) were included. The node size represents the scale of taxon abundance while edges represent the significant positive correlations between two linked nodes (*p* < 0.01). Edge width represents the size of SparCC correlation coefficient. All relative abundances and SparCC coefficient values used for network plotting were normalized via the continuous-mapping method in Cytoscape. The encircled nodes are part of the most densely connected region identified by MCODE analysis.

Using SparCC (Friedman and Alm, 2012) to examine the correlations between the skin bacterial taxa in high- and low-altitude samples, we observed an altitude-associated clustering pattern (Supplementary Figure S8, *p* < 0.01). Most high altitude associated taxa were clustered together and separated from low altitude associated taxa. The correlation between high-altitude taxa was of primary interest, so we computed a new network on only the 32 most abundant high altitude specific taxa (across humans and pigs) identified by LEfSe. Among these 32 taxa, we observed significant positive correlations (*p* < 0.01), as shown in **Figure 5**. Topological structure analysis by MCODE (a Cytoscape app) identified the most densely connected region, comprised of five strongly correlated taxa (*Arthrobacter, Paenibacillus, Carnobacterium, Desemzia*, and an unclassified genus in family *Cellulomonadaceae*; see **Figure 5**, circled area). Interestingly, all of these taxa were present in both humans and pigs.

## Discussion

Like the gut microbiome, the skin microbiome has been connected to several health and disease states in both animals and humans. However, unlike the gut microbiome, little work has been done to assess the effect of altitude on the skin microbiome. Therefore, we present here one of the first comprehensive studies on the skin microbiome of humans at low and high altitudes. We also assess the skin microbiomes of pigs living at low and high altitudes, as the similarity of pig skin to human skin makes it a potentially useful laboratory model for further skin microbiome studies.

### Low Diversity Skin Microbial Communities Are Associated with High Altitude

In both humans and pigs at high altitude, skin microbiome diversity was significantly lower, which we hypothesize is due to high-altitude selection against specific bacterial taxa. Similar observations have been reported in lake water; the diversity of Bacterioplankton in four high-altitude Tibetan freshwater lakes was only one-fifth of the Bacterioplankton diversity in seven low-altitude lakes (Xing et al., 2009). Several factors may influence bacterial diversity at high altitude; for example, the higher UVR intensity at high altitude may reduce bacterial community diversity (Supplementary Figure S2). Other environmental factors such as temperature and oxygen concentration may also contribute to the altitude-associated differences in skin microbiome diversity and structure; however, these factors are all correlated and it is difficult to parse their individual roles in shaping the skin microbiome.

A recent study similarly studied the effects of high-altitude living on the microbiome of Tibetans and Hans, but unlike our study, focused on the gut microbiome (Li and Zhao, 2015). Similar to our results, Li and Zhao observed remarkable microbiome differences between humans living at high and low altitude. Specifically, high-altitude living was associated with higher relative abundances of Firmicutes, while low-altitude living was associated with higher abundances of Bacteroidetes. While such differences may be attributable to the extreme environment in Tibet, it is important to note that the dietary habits of the Tibetans (higher fat content) differ from those of the Hans; additionally, these two races differ genetically (adaptive evolution), both of which may also contribute to the differences in microbiome composition. Unlike our study, there were no differences in alpha diversity associated with high-altitude living. This inconsistency is almost certainly due to the different ecosystems studied: the gut does not have direct contact with certain factors in the outside environment, such as intense UVR exposure, while the skin microbiome is exposed directly to the high-altitude environment and subjected to constant challenges.

Recently, Wolz et al. (2017) examined the effects of host species and environment on the skin microbiome of Plethodontid salamanders. Consistently, they found that environment has a stronger influence than species on the skin microbiome of salamanders. However, unlike our data, they found increased alpha diversity with elevation. The discrepancy in alpha diversity between the two studies might be mainly due to different species and environment. Wolz et al. (2017) sampled the skins of salamanders along a range of 700–1000 masl, which is considered as the low altitude in our study. The high-altitude environment in this study is much higher, ranging from 3750 to 3861 masl.

### The Potential Effect of Co-housing on Skin Microbiome

In our study, we observed that the skin microbiomes of high-altitude pigs were more similar to the skin microbiomes of high-altitude humans (both Tibetans and Hans) than to the skin microbiomes of pigs at a low altitude (**Figure 3E** and Supplementary Figure S3). This indicates a convergent evolution of the skin microbiome as a result of adaptation to the extreme highland environment, as supported by the PERMANOVA analysis showing the dominant effect of altitude on bacterial community diversity. Nevertheless, this observation may be due to shared living between Tibetans and Tibetan pigs. In Tibet, Tibetans live in vintage, stone brick, two-story houses; the second floor is reserved solely for human use, while part of the ground floor is used to raise Tibetan pigs. Therefore, the Tibetans and their pigs on high altitude share relatively common living environment (e.g., same house but different floors). This explains, at least in part, why the skin microbiota of Tibetan pigs were more similar to those of HAT than to HAH (Supplementary Figure S5).

### Identification of a Small “Core” Skin Microbiome Shared between High-Altitude Humans and High-Altitude Pigs

Further analyses revealed five “core” bacterial taxa (*Arthrobacter, Paenibacillus, Carnobacterium*, and two unclassified genera in families *Cellulomonadaceae* and *Xanthomonadaceae*) enriched in both humans and pigs at high altitude. Additionally, *Erwinia* was the most common genus detected on the skin of humans living at high altitude and was among the top three taxa detected on the skin of high-altitude pigs. Interestingly, most of these taxonomic groups have been reported as extremophiles isolated from high-altitude areas or similar environments, suggesting that the presence of these taxa on the skin of high-altitude humans and pigs but not on low-altitude humans and pigs is directly due to exposure to the high-altitude environment.

*Arthrobacter* spp. are widespread soil bacteria, and several species have been isolated from extreme environments including high-altitude mountains (*Arthrobacter alpinus* from alpine soil) (Zhang et al., 2010), high-altitude cold desert (Leh Ladakh of India and Xinjiang desert of China) (Yadav et al., 2015; Yu et al., 2015), and Antarctic soil (Dsouza et al., 2015). These taxa are characterized by broad adaptability and have been described as cold-tolerant, desiccation-tolerant, and salt-tolerant. *Arthrobacter* does not appear to be a normal skin commensal on human or animal as it has been reported only rarely on human (*Arthrobacter scleromae* and *Arthrobacter oxydans*; Huang et al., 2005) or animal skin (*Arthrobacter equi* in horses; Yassin et al., 2011), suggesting that its notable abundance on the skin of both high-altitude humans and high-altitude pigs is directly due to the high-altitude lifestyle. Further studies have to reveal whether *Arthrobacter* is simply a commensal picked up from the environment or if it plays a specific role in protecting the skin against the harsh environment found at high altitude.

Like *Arthrobacter, Paenibacillus* is commonly found in soil, and is often associated with plant roots (Kim et al., 2015; Zhang et al., 2015). Interestingly, *Paenibacillus* spp. can produce a wide variety of exopolysaccharides (EPSs), which in other species have been report to have significant antimicrobial activity and the ability to improve skin hydration (Liang et al., 2014), a potentially useful advantage for high-altitude skin, which is prone to dehydration. Additionally, EPS from three *Paenibacillus* species (*Paenibacillus polymyxa* SQR-21, *Paenibacillus* sp. TKU023, and *P. polymyxa* EJS-3) has been reported to have antioxidative properties (Raza et al., 2011; Wang et al., 2011; Liu et al., 2012). In animals and plants, antioxidants have proven critical for high-altitude adaptation (Sharma et al., 2015; Zhanga et al., 2015); therefore, the presence of *Paenibacillus* on the skin of high-altitude humans and pigs may indeed be an adaptive advantage for protecting skin against hypoxia, UV radiation, and drought typical of the Tibetan Plateau.

*Carnobacterium* spp. are extremophiles that have been isolated from environments including Siberian permafrost (Leonard et al., 2013) and an Antarctic pond (Snauwaert et al., 2013). Interestingly, two permafrost isolates (*Carnobacterium gilichinskyi* and *Carnobacterium funditum*) grew under the extreme conditions of simulated Martian soil, characterized by low temperature (0°C), low-pressure (7 mPa), and CO_2_-enriched anoxic conditions (Nicholson et al., 2013). Because *Carnobacterium* has been rarely reported in human or animal skin samples, we hypothesize that it’s presence on the skin of high-altitude humans and animals in our study is due to exposure to the high-altitude environment and that these bacteria may be providing some skin-protective effect at high altitude. Nevertheless, further studies are needed to confirm this hypothesis.

*Erwinia* spp. have been recognized as plant pathogens since the 1930s (Elrod, 1942) and can infect a wide range of plants including cucumbers, melons, squashes, and many other (Piqué et al., 2015). In humans, *Erwinia* was first isolated from a wound in 1966 (Von Graevenitz and Strouse, 1966) and the following year observed again in infected skin (Slotnick and Tulman, 1967). *Erwinia* has also been isolated from grain mill dust and from human and animal sources (Dutkiewicz, 1976). Interestingly, it has been shown that *Erwinia* spp. can survive in and be transmitted by plant insects (Campillo et al., 2015; Ordax et al., 2015). Unlike other taxa detected on the skin of high-altitude pigs and humans, *Erwinia* does not appear to be particularly adapted to high-altitude environments; however, it may be present in and around the barley fields grown by Tibetans. Therefore, the role of this specific bacterial genus in host adaptation to a high-altitude environment needs further validation.

### Study Limitations

There are some limitations to our study. First, we only collected skin samples from Tibetans in two villages 6 miles away from each other and it is impossible to say how representative these two communities are of the Tibetan population as a whole. However, these villages have been home to Tibetans for many generations, and the Tibetans living there engage in typical Tibetan living and hygiene practices. Nevertheless, larger-scale studies sampling a variety of Tibetan communities will serve to validate (or refute) our results. Another limitation to our study is that some of the Tibetans shared the same living house with their pigs. As it has been well-described that animals and humans sharing homes also share microbiomes (Song et al., 2013; Lax et al., 2014), the “core” skin microbiome we observe in high-altitude pigs and humans may be due to shared living space rather than high-altitude. Therefore, further studies examining high-altitude pigs not sharing living spaces with humans will help determine whether a core skin microbiome is in fact due to high-altitude living. A final limitation of our study is the usage of 16S variable region 4 primers, which are known to discriminate against some skin-associated taxa (Meisel et al., 2016). Nevertheless, our usage of the V4 primers may be the reason why we were able to detect soil and other important environmental microbes on the skin, which may have been low abundance or undetected if using primers more specific for skin microbes.

## Conclusion

In conclusion, we utilized 16S rRNA gene sequencing to characterize the skin microbiomes of humans and pigs living in high- and low-altitude areas of China, identifying significant differences in both diversity and composition and a core community found in high-altitude humans and pigs. These results lay the groundwork for further studies exploring the possibility of high-altitude adaptation of beneficial skin-associated microbial communities in humans and other mammals.

## Data Accessibility

Sequence data accession number: SRP065099, Sequence Read Archive (SRA) in NCBI.

## Author Contributions

YL and BZ conceived and designed the experiments. BZ, WG, SZ, YH, XuY, LF, KL, XiY, GC, LJ, SS, and JY contributed to sampling. BZ, JZ, WG, JT, FK, XS, and RN performed the experiments and analyzed data. BZ, JZ, SM, and YL wrote the paper.

## Conflict of Interest Statement

The authors declare that the research was conducted in the absence of any commercial or financial relationships that could be construed as a potential conflict of interest.

## References

[B1] Bastos M. C. F. Ceotto H. Coelho M. L. V. Nascimento J. S. (2009). Staphylococcal antimicrobial peptides: relevant properties and potential biotechnological applications. *Curr. Pharmaceut. Biotechnol.* 10 38–61. 10.2174/138920109787048580 19149589

[B2] Beall C. M. (2006). Andean, Tibetan, and Ethiopian patterns of adaptation to high-altitude hypoxia. *Integr. Compar. Biol.* 46 18–24. 10.1093/icb/icj004 21672719

[B3] Campillo T. Luna E. Portier P. Fischer-Le Saux M. Lapitan N. Tisserat N. A. (2015). *Erwinia iniecta* sp. nov., isolated from Russian wheat aphid (*Diuraphis noxia*). *Int. J. Syst. Evolut. Microbiol.* 65 3625–3633. 10.1099/ijsem.0.000466 26198254

[B4] Caporaso J. G. Kuczynski J. Stombaugh J. Bittinger K. Bushman F. D. Costello E. K. (2010). QIIME allows analysis of high-throughput community sequencing data. *Nat. Methods* 7 335–336. 10.1038/nmeth.f.303 20383131 PMC3156573

[B5] Caporaso J. G. Lauber C. L. Walters W. A. Berg-Lyons D. Lozupone C. A. Turnbaugh P. J. (2011). Global patterns of 16S rRNA diversity at a depth of millions of sequences per sample. *Proc. Natl. Acad. Sci. U.S.A.* 108(Suppl. 1) 4516–4522. 10.1073/pnas.1000080107 20534432 PMC3063599

[B6] Dsouza M. Taylor M. W. Turner S. J. Aislabie J. (2015). Genomic and phenotypic insights into the ecology of Arthrobacter from Antarctic soils. *BMC Genomics* 16:36 10.1186/s12864-015-1220-2 PMC4326396 25649291

[B7] Dutkiewicz J. (1976). Studies on endotoxin of *Erwinia herbicola* and their biological activity. *Zentralbl. Bakteriol. Orig. A* 236 487–508. 1015031

[B8] Edgar R. C. Haas B. J. Clemente J. C. Quince C. Knight R. (2011). UCHIME improves sensitivity and speed of chimera detection. *Bioinformatics* 27 2194–2200. 10.1093/bioinformatics/btr381 21700674 PMC3150044

[B9] Elrod R. P. (1942). The *Erwinia*-coliform relationship. *J. Bacteriol.* 44 433–440. 16560581 10.1128/jb.44.4.433-440.1942 PMC373693

[B10] Fierer N. Hamady M. Lauber C. L. Knight R. (2008). The influence of sex, handedness, and washing on the diversity of hand surface bacteria. *Proc. Natl. Acad. Sci. U.S.A.* 105 17994–17999. 10.1073/pnas.0807920105 19004758 PMC2584711

[B11] Friedman J. Alm E. J. (2012). Inferring correlation networks from genomic survey data. *PLOS Comput. Biol.* 8:e1002687 10.1371/journal.pcbi.1002687 PMC3447976 23028285

[B12] Gallo R. L. Nakatsuji T. (2011). Microbial symbiosis with the innate immune defense system of the skin. *J. Invest. Dermatol.* 131 1974–1980. 10.1038/jid.2011.182 21697881 PMC3174284

[B13] Grice E. A. Kong H. H. Conlan S. Deming C. B. Davis J. Young A. C. (2009). Topographical and temporal diversity of the human skin microbiome. *Science* 324 1190–1192. 10.1126/science.1171700 19478181 PMC2805064

[B14] Grice E. A. Snitkin E. S. Yockey L. J. Bermudez D. M. Program N. C. S. Liechty K. W. (2010). Longitudinal shift in diabetic wound microbiota correlates with prolonged skin defense response. *Proc. Natl. Acad. Sci. U.S.A.* 107 14799–14804. 10.1073/pnas.1004204107 20668241 PMC2930465

[B15] He Z. Zhang H. Gao S. Lercher M. J. Chen W.-H. Hu S. (2016). Evolview v2: an online visualization and management tool for customized and annotated phylogenetic trees. *Nucleic Acids Res.* 44 W236–W241. 10.1093/nar/gkw370 27131786 PMC4987921

[B16] Huang Y. Zhao N. He L. Wang L. Liu Z. You M. (2005). *Arthrobacter scleromae* sp. nov. Isolated from human clinical specimens. *J. Clin. Microbiol.* 43 1451–1455. 10.1128/JCM.43.3.1451-1455.2005 15750131 PMC1081264

[B17] Jablonski N. G. Chaplin G. (2010). Human skin pigmentation as an adaptation to UV radiation. *Proc. Natl. Acad. Sci. U.S.A.* 107(Suppl. 2) 8962–8968. 10.1073/pnas.0914628107 20445093 PMC3024016

[B18] Kim T.-S. Han J.-H. Joung Y. Kim S. B. (2015). *Paenibacillus oenotherae* sp. nov. and *Paenibacillus hemerocallicola* sp. nov., isolated from the roots of herbaceous plants. *Int. J. Syst. Evol. Microbiol.* 65 2717–2725. 10.1099/ijs.0.000329 25977281

[B19] Kueneman J. G. Parfrey L. W. Woodhams D. C. Archer H. M. Knight R. McKenzie V. J. (2014). The amphibian skin-associated microbiome across species, space and life history stages. *Mol. Ecol.* 23 1238–1250. 10.1111/mec.12510 24171949

[B20] Lai Y. Cogen A. L. Radek K. A. Park H. J. MacLeod D. T. Leichtle A. (2010). Activation of TLR2 by a small molecule produced by *Staphylococcus epidermidis* increases antimicrobial defense against bacterial skin infections. *J. Invest. Dermatol.* 130 2211–2221. 10.1038/jid.2010.123 20463690 PMC2922455

[B21] Larsen A. M. Bullard S. A. Womble M. Arias C. R. (2015). Community structure of skin microbiome of Gulf Killifish, *Fundulus grandis*, is driven by seasonality and not exposure to oiled sediments in a Louisiana Salt Marsh. *Microb. Ecol.* 70 534–544. 10.1007/s00248-015-0578-7 25704317

[B22] Lax S. Smith D. P. Hampton-Marcell J. Owens S. M. Handley K. M. Scott N. M. (2014). Longitudinal analysis of microbial interaction between humans and the indoor environment. *Science* 345 1048–1052. 10.1126/science.1254529 25170151 PMC4337996

[B23] Leonard M. T. Panayotova N. Farmerie W. G. Triplett E. W. Nicholson W. L. (2013). Complete genome sequence of *Carnobacterium gilichinskyi* strain WN1359(T) (DSM 27470T). *Genome Announc.* 1:e00985-13. 10.1128/genomeA.00985-13 PMC3869332 24285647

[B24] Li L. Zhao X. (2015). Comparative analyses of fecal microbiota in Tibetan and Chinese Han living at low or high altitude by barcoded 454 pyrosequencing. *Sci. Rep.* 5:14682 10.1038/srep14682 PMC4595765 26443005

[B25] Liang T.-W. Wu C.-C. Cheng W.-T. Chen Y.-C. Wang C.-L. Wang I. L. (2014). Exopolysaccharides and antimicrobial biosurfactants produced by *Paenibacillus macerans* TKU029. *Appl. Biochem. Biotechnol.* 172 933–950. 10.1007/s12010-013-0568-5 24122708 PMC3918387

[B26] Liu J. Luo J. Ye H. Zeng X. (2012). Preparation, antioxidant and antitumor activities in vitro of different derivatives of levan from endophytic bacterium *Paenibacillus polymyxa* EJS-3. *Food Chem. Toxicol.* 50 767–772. 10.1016/j.fct.2011.11.016 22142695

[B27] Meisel J. S. Hannigan G. D. Tyldsley A. S. SanMiguel A. J. Hodkinson B. P. Zheng Q. (2016). Skin microbiome surveys are strongly influenced by experimental design. *J. Invest. Dermatol.* 136 947–956. 10.1016/j.jid.2016.01.016 26829039 PMC4842136

[B28] Nakatsuji T. Chiang H.-I. Jiang S. B. Nagarajan H. Zengler K. Gallo R. L. (2013). The microbiome extends to subepidermal compartments of normal skin. *Nat. Commun.* 4 1431–1431. 10.1038/ncomms2441 23385576 PMC3655727

[B29] Nicholson W. L. Krivushin K. Gilichinsky D. Schuerger A. C. (2013). Growth of *Carnobacterium* spp. from permafrost under low pressure, temperature, and anoxic atmosphere has implications for Earth microbes on Mars. *Proc. Natl. Acad. Sci. U.S.A.* 110 666–671. 10.1073/pnas.1209793110 23267097 PMC3545801

[B30] Olsen C. M. Wilson L. F. Green A. C. Bain C. J. Fritschi L. Neale R. E. (2015). Cancers in Australia attributable to exposure to solar ultraviolet radiation and prevented by regular sunscreen use. *Aust. N. Z. J. Public Health* 39 471–476. 10.1111/1753-6405.12470 26437734 PMC4606762

[B31] Ordax M. Piquer-Salcedo J. E. Santander R. D. Sabater-Muñoz B. Biosca E. G. López M. M. (2015). Medfly *Ceratitis capitata* as potential vector for fire blight pathogen *Erwinia amylovora*: survival and transmission. *PLOS ONE* 10:e0127560 10.1371/journal.pone.0127560 PMC4433354 25978369

[B32] Piqué N. Miñana-Galbis D. Merino S. Tomás J. M. (2015). Virulence factors of *Erwinia amylovora*: a review. *Int. J. Mol. Sci.* 16 12836–12854. 10.3390/ijms160612836 26057748 PMC4490474

[B33] Raza W. Makeen K. Wang Y. Xu Y. Qirong S. (2011). Optimization, purification, characterization and antioxidant activity of an extracellular polysaccharide produced by *Paenibacillus polymyxa* SQR-21. *Bioresour. Technol.* 102 6095–6103. 10.1016/j.biortech.2011.02.033 21392978

[B34] Rodrigues Hoffmann A. Patterson A. P. Diesel A. Lawhon S. D. Ly H. J. Stephenson C. E. (2014). The skin microbiome in healthy and allergic dogs. *PLOS ONE* 9:e83197 10.1371/journal.pone.0083197 PMC3885435 24421875

[B35] Segata N. Izard J. Waldron L. Gevers D. Miropolsky L. Garrett W. S. (2011). Metagenomic biomarker discovery and explanation. *Genome Biol.* 12:R60 10.1186/gb-2011-12-6-r60 PMC3218848 21702898

[B36] Sharma P. Suryakumar G. Singh V. Misra K. Singh S. B. (2015). In vitro antioxidant profiling of seabuckthorn varieties and their adaptogenic response to high altitude-induced stress. *Int. J. Biometeorol.* 59 1115–1126. 10.1007/s00484-014-0925-2 25384585

[B37] Slotnick I. J. Tulman L. (1967). A human infection caused by an *Erwinia* species. *Am. J. Med.* 43 147–150. 10.1016/0002-9343(67)90157-X 4951415

[B38] Smeekens S. P. Huttenhower C. Riza A. van de Veerdonk F. Zeeuwen P. L. J. M. Schalkwijk J. (2014). Skin microbiome imbalance in patients with STAT1/STAT3 defects impairs innate host defense responses. *J. Innate Immun.* 6 253–262. 10.1159/000351912 23796786 PMC4045018

[B39] Snauwaert I. Hoste B. Bruyne K. D. Peeters K. Vuyst L. D. Willems A. (2013). *Carnobacterium iners* sp. nov., a psychrophilic, lactic acid-producing bacterium from the littoral zone of an Antarctic pond. *Int. J. Syst. Evol. Microbiol.* 63(Pt 4) 1370–1375. 10.1099/ijs.0.042861-0 22798642

[B40] Song S. J. Lauber C. Costello E. K. Lozupone C. A. Humphrey G. Berg-Lyons D. (2013). Cohabiting family members share microbiota with one another and with their dogs. *eLife* 2:e00458 10.7554/eLife.00458 PMC3628085 23599893

[B41] Staudinger T. Pipal A. Redl B. (2011). Molecular analysis of the prevalent microbiota of human male and female forehead skin compared to forearm skin and the influence of make-up. *J. Appl. Microbiol.* 110 1381–1389. 10.1111/j.1365-2672.2011.04991.x 21362117

[B42] Summerfield A. Meurens F. Ricklin M. E. (2015). The immunology of the porcine skin and its value as a model for human skin. *Mol. Immunol.* 66 14–21. 10.1016/j.molimm.2014.10.023. 25466611

[B43] Von Graevenitz A. Strouse A. (1966). Isolation of *Erwinia* spp. from human sources. *Antonie van Leeuwenhoek* 32 429–430. 10.1007/BF02097494 5297392

[B44] Walke J. B. Becker M. H. Loftus S. C. House L. L. Cormier G. Jensen R. V. (2014). Amphibian skin may select for rare environmental microbes. *ISME J.* 8 2207–2217. 10.1038/ismej.2014.77 24858782 PMC4992085

[B45] Wang C.-L. Huang T.-H. Liang T.-W. Fang C.-Y. Wang S.-L. (2011). Production and characterization of exopolysaccharides and antioxidant from *Paenibacillus* sp. TKU023. *N. Biotechnol.* 28 559–565. 10.1016/j.nbt.2011.03.003 21402186

[B46] Wolz C. R. M. Yarwood S. A. Evan H. Campbell Grant Fleischer R. C. Lips K. R. (2017). Effects of host species and environment on the skin microbiome of Plethodontid salamanders. *J. Anim. Ecol.* 10.1111/1365-2656.12726 [Epub ahead of print]. 28682480

[B47] Xing P. Hahn M. W. Wu Q. L. (2009). Low taxon richness of bacterioplankton in high-altitude lakes of the eastern tibetan plateau, with a predominance of bacteroidetes and *Synechococcus* spp. *Appl. Environ. Microbiol.* 75 7017–7025. 10.1128/AEM.01544-09 19767472 PMC2786500

[B48] Yadav A. N. Sachan S. G. Verma P. Tyagi S. P. Kaushik R. Saxena A. K. (2015). Culturable diversity and functional annotation of psychrotrophic bacteria from cold desert of Leh Ladakh (India). *World J. Microbiol. Biotechnol.* 31 95–108. 10.1007/s11274-014-1768-z 25371316

[B49] Yassin A. F. Spröer C. Siering C. Hupfer H. Schumann P. (2011). *Arthrobacter equi* sp. nov., isolated from veterinary clinical material. *Int. J. Syst. Evol. Microbiol.* 61(Pt 9) 2089–2094. 10.1099/ijs.0.026690-0 20870884

[B50] Yu X.-Y. Zhang L. Ren B. Yang N. Liu M. Liu X.-T. (2015). *Arthrobacter liuii* sp. nov., resuscitated from Xinjiang desert soil. *Int. J. Syst. Evol. Microbiol.* 65(Pt 3) 896–901. 10.1099/ijs.0.000037 25525122

[B51] Zhang C. Li M. Xu X. Liu N. (2015). Effects of carbon nanotubes on atrazine biodegradation by *Arthrobacter* sp. *J. Hazard. Mater.* 287 1–6. 10.1016/j.jhazmat.2015.01.039 25621828

[B52] Zhang D.-C. Schumann P. Liu H.-C. Xin Y.-H. Zhou Y.-G. Schinner F. (2010). *Arthrobacter alpinus* sp. nov., a psychrophilic bacterium isolated from alpine soil. *Int. J. Syst. Evol. Microbiol.* 60(Pt 9) 2149–2153. 10.1099/ijs.0.017178-0 19880631

[B53] Zhanga Y. Liang S. He J. Bai Y. Niu Y. Tang X. (2015). Oxidative stress and antioxidant status in a lizard *Phrynocephalus vlangalii* at different altitudes or acclimated to hypoxia. *Comp. Biochem. Physiol. A Mol. Integr. Physiol.* 190 9–14. 10.1016/j.cbpa.2015.08.013 26310105

