# Supplementary material for: High-Altitude Living Shapes the Skin Microbiome in Humans and Pigs
Source: Front Microbiol. 2017 Oct 6;8:1929. doi: 10.3389/fmicb.2017.01929 (PMC5635199; doi:10.3389/fmicb.2017.01929)
Supplement: Supplementary file 1 [file Data_Sheet_1.pdf]

Supplementary Material

High-altitude living shapes the skin microbiome in humans and pigs

Bo Zeng[1,6], Jiangchao Zhao[3,6], Wei Guo[1,6]

*** Correspondence:** Ying Li: yingli@sicau.edu.cn

Supplementary Figures

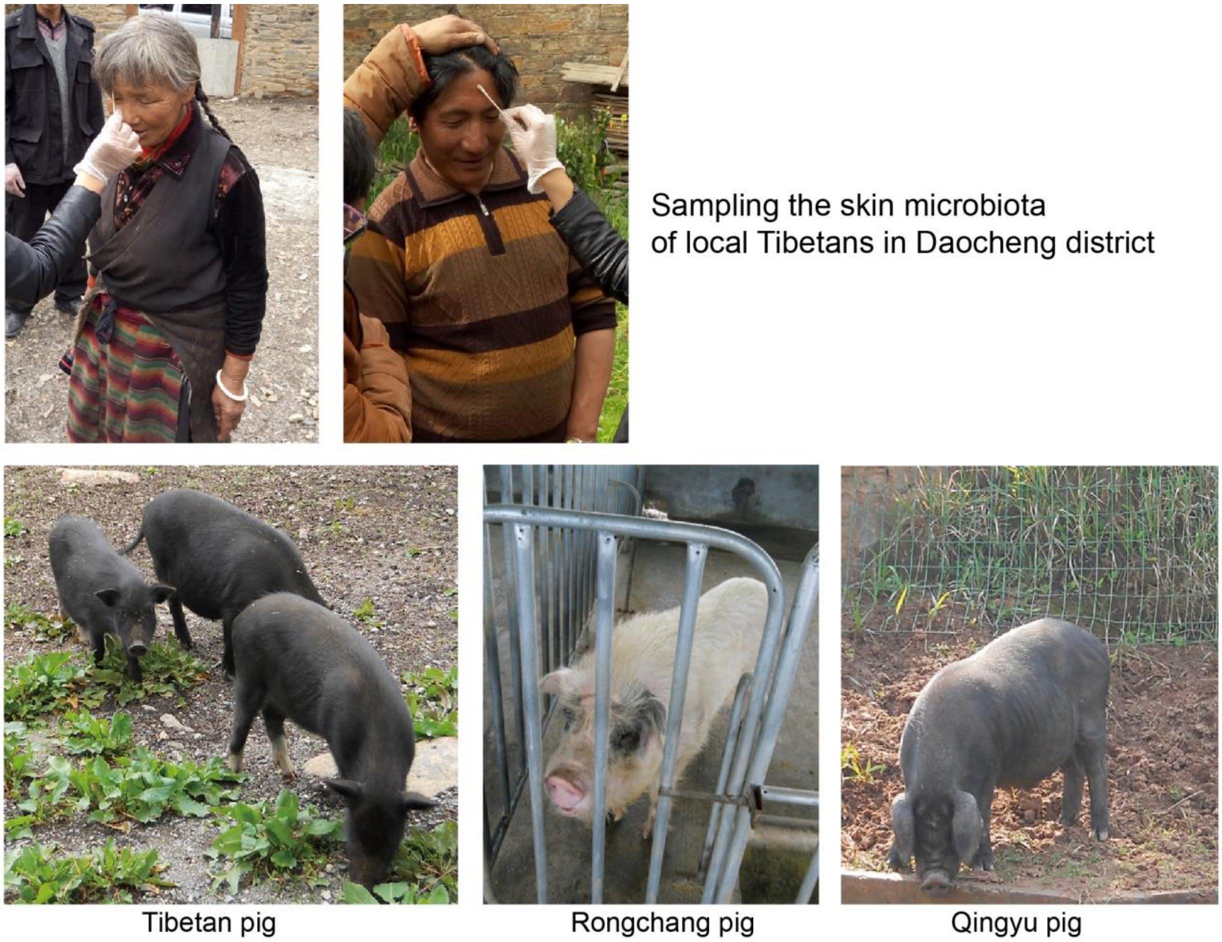

Figure S1. Photo examples of human forehead skin sampling and three pig breeds sampled in this study.

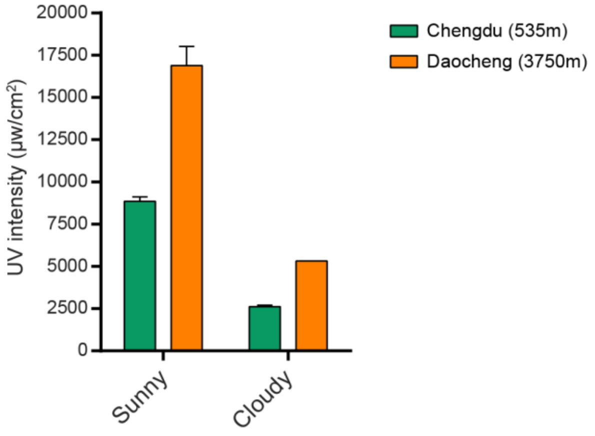

Figure S2. Comparison of the ultraviolet intensity between sampling districts of high and low altitude.

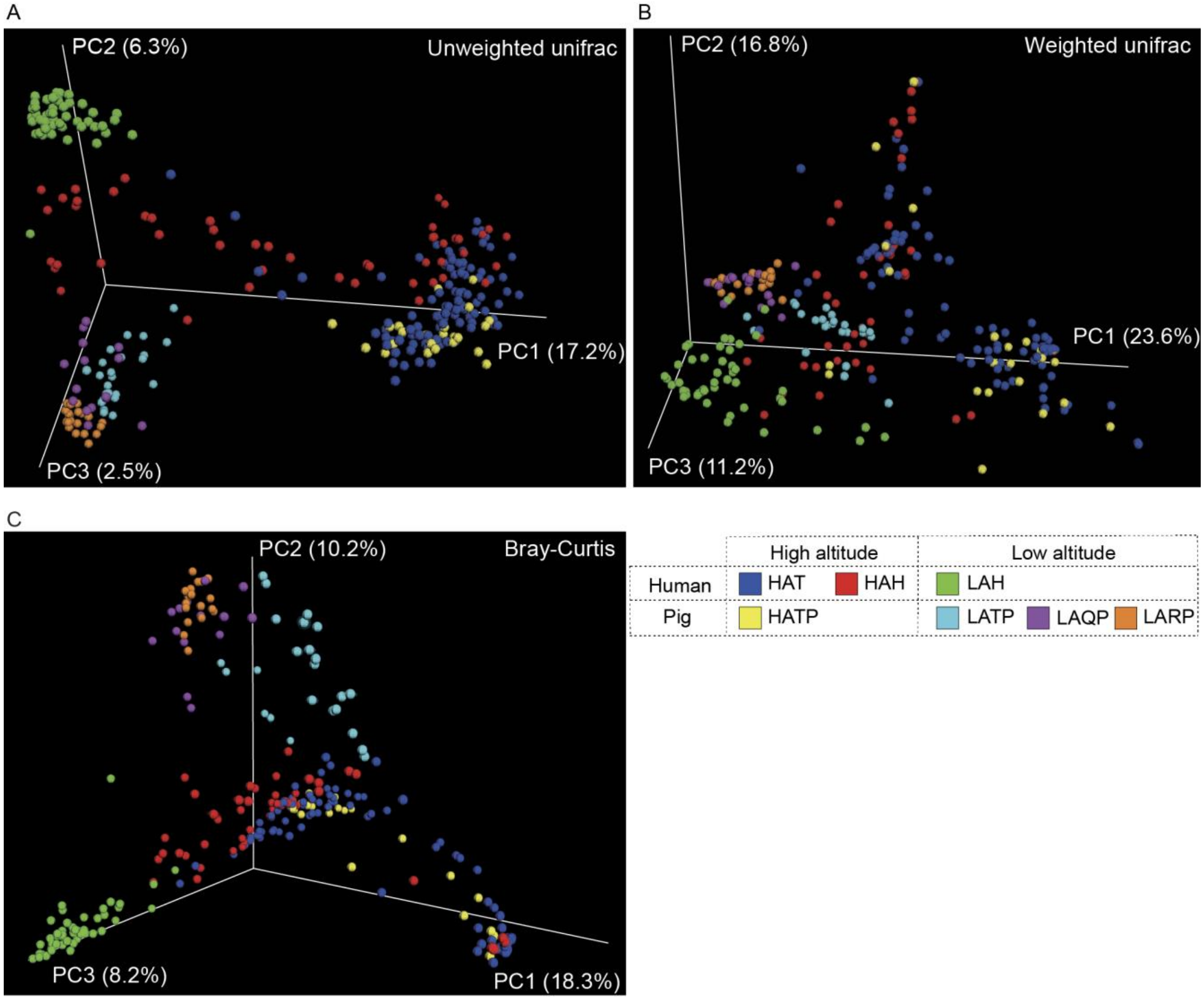

Figure S3. Jackknifed beta diversity analysis of skin microbiota for both human and pig groups. **A:** Principle Coordinate Analysis (PCoA) of skin microbiota based on unweighted Unifrac distances. **B:** PCoA plots based on weighted Unifrac distances. **C:** PCoA plots based on Bray-Curtis distances.

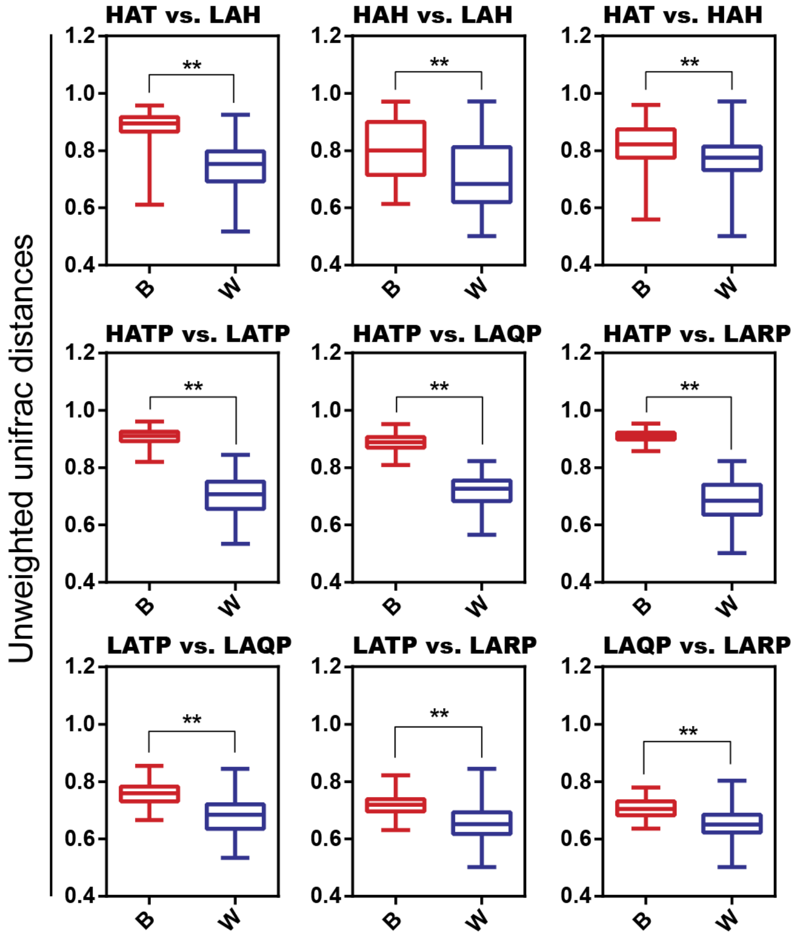

Figure S4. Comparison of unweighted unifrac distances between paired groups. Unweighted unifrac distances between each pair of samples were calculated. Significance tests between groups were performed by comparing between and within distance values of each paired group. **B**: distances between two comparing groups. **W**: distances within these two groups. **: p <0.01, Mann-Whitney U test.

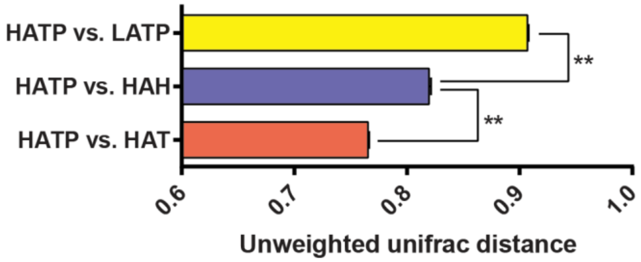

Figure S5. Comparison of unweighted unifrac distances between humans and pigs. ** p<0.01, Mann-Whitney U test.

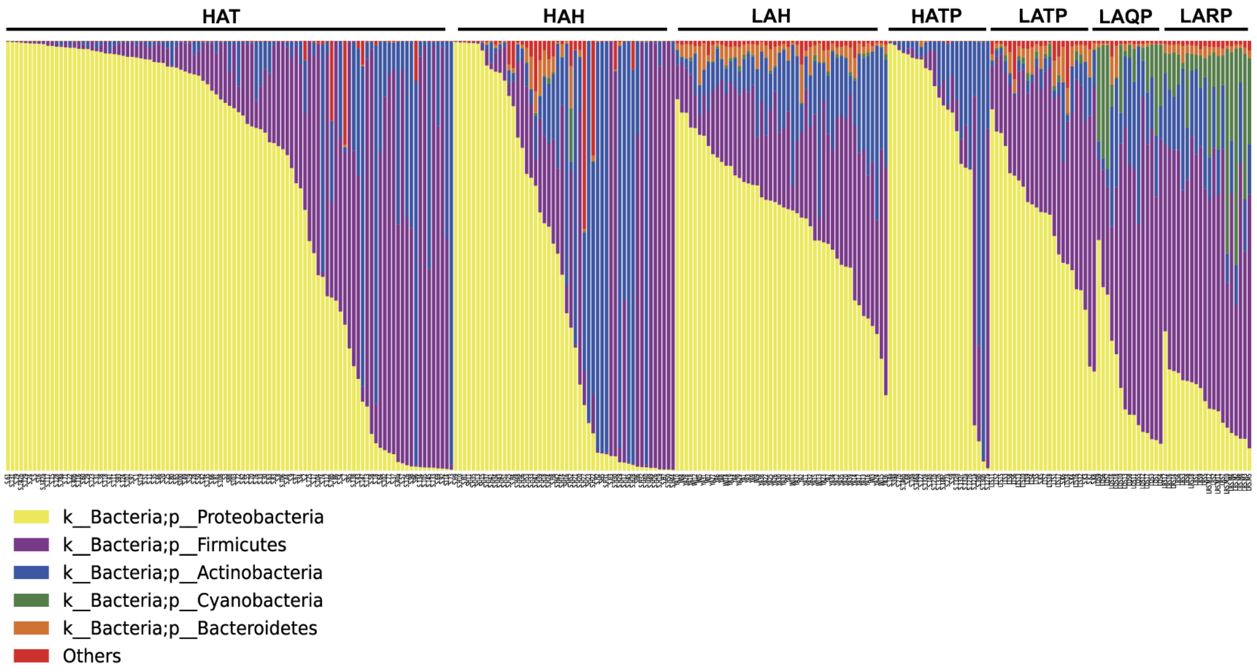

Figure S6. Composition structure of skin bacteria summarized at phylum level.

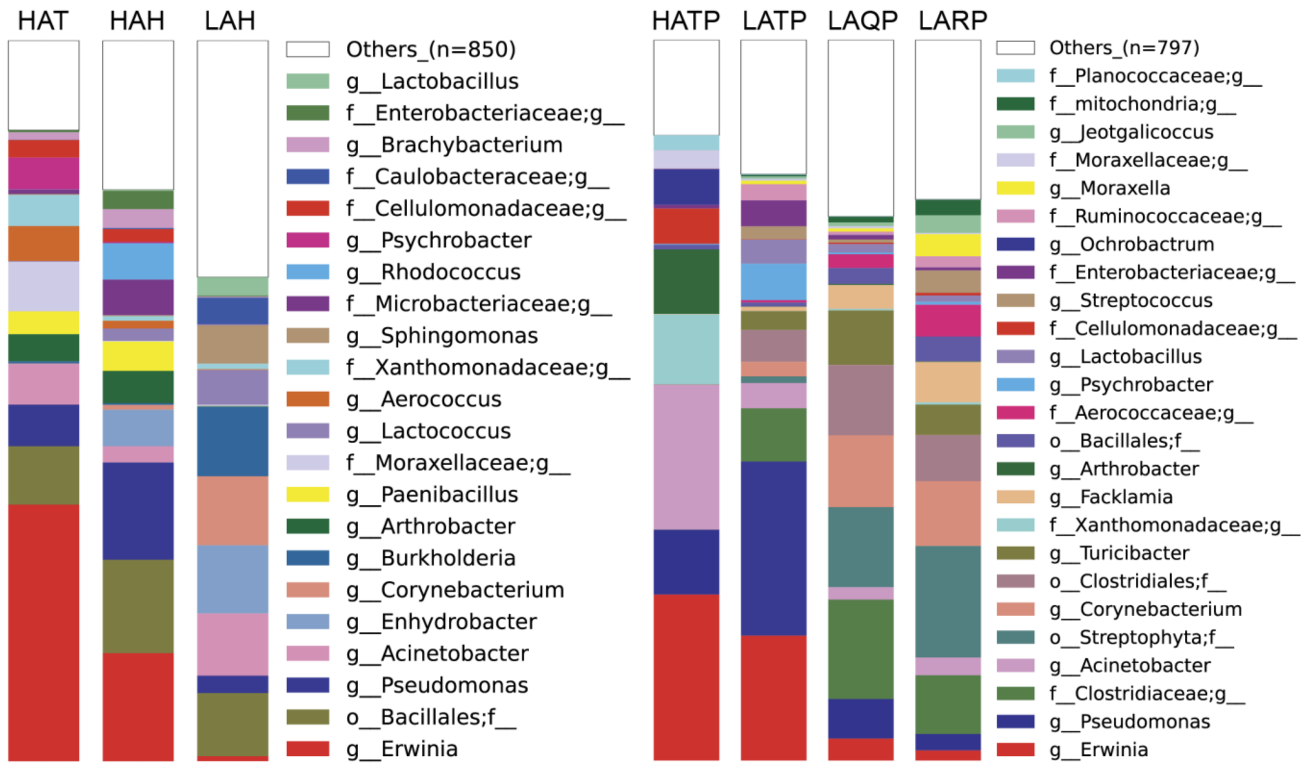

Figure S7. Skin bacteria composition of high- and low-altitude groups. Each bar represents the average relative abundance of each bacterial taxon within a sample group.

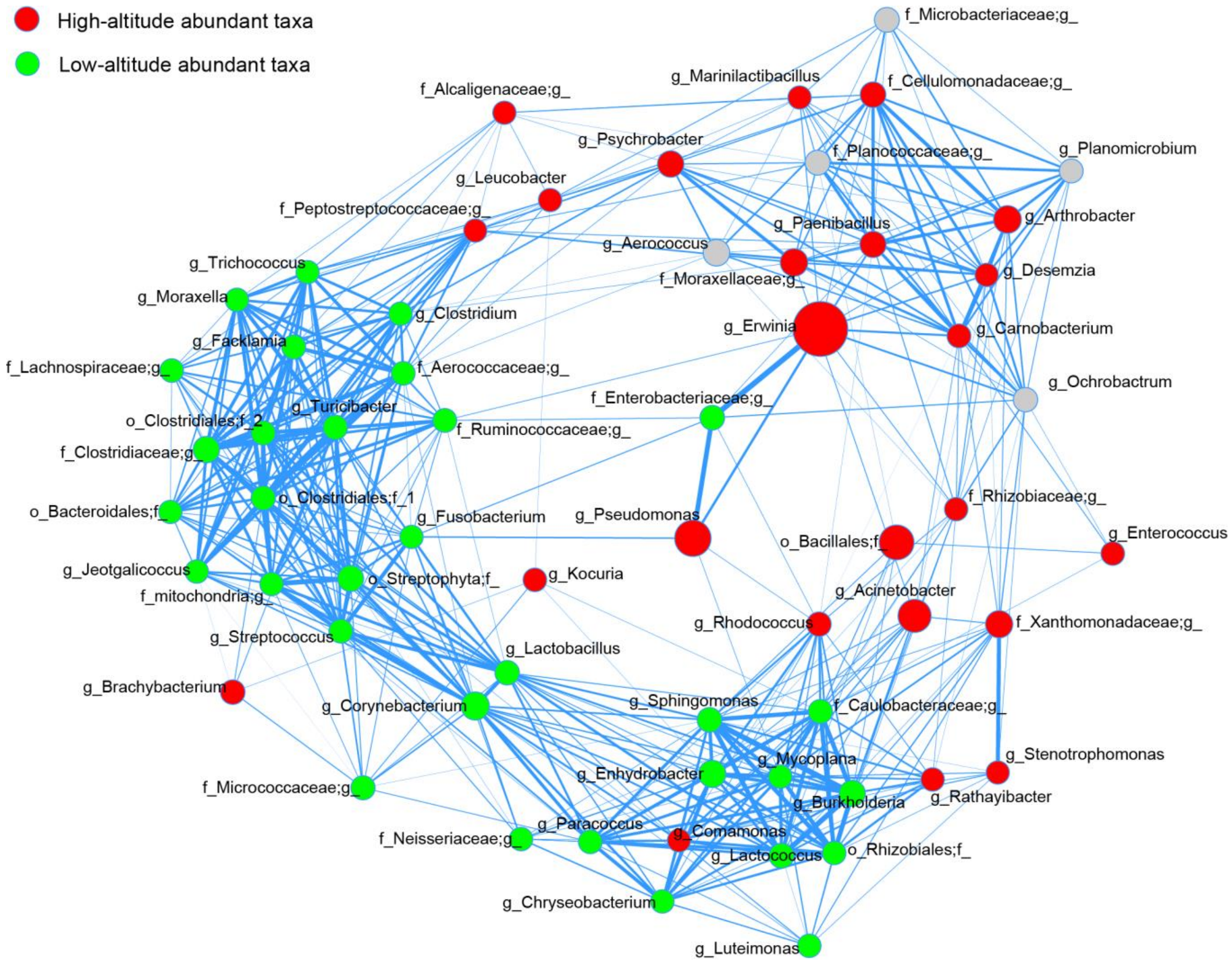

Figure S8. Correlation network of the abundant skin bacteria. The relative abundance matrix data of 61 most abundant skin taxa were input for SparCC correlation analysis and visualized by customized cytoscape network. Data of all samples (both human and pig) were included. Nodes (labeled with skin taxa names) were colored according to the significance test results of LEfSe which comparing between high- and low altitude groups (All high-altitude samples *vs.* all low-altitude samples). Node size represents the scale of taxa abundance. Edges represent the significant positive correlations were found between two linked nodes (p <0.01). Edge width represents the size of SparCC correlation coefficient. (All values of taxa relative abundance and SparCC coefficient were normalized by continuous mapping while plotting in Cytoscape).
