# Supplementary material for: High-Altitude Living Shapes the Skin Microbiome in Humans and Pigs
Source: Front Microbiol. 2017 Oct 6;8:1929. doi: 10.3389/fmicb.2017.01929 (PMC5635199; doi:10.3389/fmicb.2017.01929)
Supplement: Supplementary file 2 [file Data_Sheet_2.pdf]

# *Supplementary Material*

# High-altitude living shapes the skin microbiome in humans and pigs

Bo Zeng[1,6], Jiangchao Zhao[3,6], Wei Guo[1,6]
*** Correspondence:** Ying Li: yingli@sicau.edu.cn

## 1. Supplementary Tables

Table S1: Sample metadata and OTU table summary.

| ID | Groups | Species | Human population / Animal breeds | Altitude | Elevation(m) | City_area | Age_year | Gender | Family_ID | Temperature | OTUs | Sequence_counts |
|---|---|---|---|---|---|---|---|---|---|---|---|---|
| S.H24 | HAH | Human | Han | high | 3750 | Daocheng | 46 | Male | N/A | 21 | 391 | 4631 |
| S.H26 | HAH | Human | Han | high | 3750 | Daocheng | 24 | Female | N/A | 21 | 82 | 5330 |
| S.H30 | HAH | Human | Han | high | 3750 | Daocheng | 28 | Male | N/A | 21 | 126 | 5850 |
| SH13 | HAH | Human | Han | high | 3750 | Daocheng | 21 | Male | c4 | 21 | 86 | 6303 |
| S.H17 | HAH | Human | Han | high | 3750 | Daocheng | 38 | Female | c5 | 21 | 67 | 7214 |
| S.H32 | HAH | Human | Han | high | 3750 | Daocheng | 36 | Female | N/A | 21 | 69 | 7356 |
| SH16 | HAH | Human | Han | high | 3750 | Daocheng | 37 | Male | c5 | 21 | 178 | 8909 |
| S.H41 | HAH | Human | Han | high | 3750 | Daocheng | 45 | Male | N/A | 21 | 166 | 9582 |
| S.H31 | HAH | Human | Han | high | 3750 | Daocheng | 60 | Male | N/A | 21 | 78 | 10343 |
| S.H19 | HAH | Human | Han | high | 3750 | Daocheng | 43 | Female | N/A | 21 | 58 | 10579 |
| S.H52 | HAH | Human | Han | high | 3750 | Daocheng | 43 | Female | N/A | 21 | 933 | 10641 |
| S.H29 | HAH | Human | Han | high | 3750 | Daocheng | 31 | Female | N/A | 21 | 291 | 11508 |
| S.H10 | HAH | Human | Han | high | 3750 | Daocheng | 37 | Male | N/A | 21 | 96 | 13043 |
| S.H49 | HAH | Human | Han | high | 3750 | Daocheng | 42 | Male | N/A | 21 | 60 | 13064 |
| S.H34 | HAH | Human | Han | high | 3750 | Daocheng | 44 | Female | c8 | 21 | 277 | 13561 |
| S.H2 | HAH | Human | Han | high | 3750 | Daocheng | 34 | Female | c1 | 21 | 145 | 14916 |
| S.H14 | HAH | Human | Han | high | 3750 | Daocheng | 37 | Female | N/A | 21 | 562 | 15712 |
| S.H27 | HAH | Human | Han | high | 3750 | Daocheng | 35 | Male | c7 | 21 | 473 | 15869 |
| SH5 | HAH | Human | Han | high | 3750 | Daocheng | 33 | Female | c2 | 21 | 90 | 16067 |
| S.H47 | HAH | Human | Han | high | 3750 | Daocheng | 51 | Male | c9 | 21 | 444 | 16109 |
| S.H45 | HAH | Human | Han | high | 3750 | Daocheng | 51 | Male | c12 | 21 | 54 | 17275 |
| S.H39 | HAH | Human | Han | high | 3750 | Daocheng | 34 | Female | c10 | 21 | 925 | 17508 |
| S.H50 | HAH | Human | Han | high | 3750 | Daocheng | 22 | Female | c13 | 21 | 86 | 17837 |
| S.H40 | HAH | Human | Han | high | 3750 | Daocheng | 45 | Female | N/A | 21 | 159 | 18070 |
| S.H25 | HAH | Human | Han | high | 3750 | Daocheng | 49 | Male | N/A | 21 | 569 | 19079 |
| S.H35 | HAH | Human | Han | high | 3750 | Daocheng | 45 | Male | c8 | 21 | 140 | 20571 |
| S.H1 | HAH | Human | Han | high | 3750 | Daocheng | 36 | Male | c1 | 21 | 148 | 21551 |
| S.H7 | HAH | Human | Han | high | 3750 | Daocheng | 35 | Female | c3 | 21 | 114 | 22922 |
| S.H8 | HAH | Human | Han | high | 3750 | Daocheng | 50 | Female | N/A | 21 | 1059 | 23507 |
| S.H33 | HAH | Human | Han | high | 3750 | Daocheng | 37 | Male | N/A | 21 | 769 | 23699 |
| SH6 | HAH | Human | Han | high | 3750 | Daocheng | 35 | Male | c3 | 21 | 136 | 23782 |
| SH11 | HAH | Human | Han | high | 3750 | Daocheng | 46 | Male | c4 | 21 | 182 | 23817 |
| SH15 | HAH | Human | Han | high | 3750 | Daocheng | 36 | Female | N/A | 21 | 359 | 24073 |
| S.H18 | HAH | Human | Han | high | 3750 | Daocheng | 31 | Female | N/A | 21 | 869 | 25691 |
| SH23 | HAH | Human | Han | high | 3750 | Daocheng | 0.5 | Female | c6 | 21 | 103 | 25715 |
| S.H36 | HAH | Human | Han | high | 3750 | Daocheng | 40 | Male | N/A | 21 | 474 | 25798 |
| S.H28 | HAH | Human | Han | high | 3750 | Daocheng | 38 | Male | c7 | 21 | 94 | 26157 |

| | | | | | | | | | | | | |
|---|---|---|---|---|---|---|---|---|---|---|---|---|
| S.H22 | HAH | Human | Han | high | 3750 | Daocheng | 24 | Female | c6 | 21 | 865 | 26308 |
| S.H43 | HAH | Human | Han | high | 3750 | Daocheng | 49 | Male | c11 | 21 | 477 | 27127 |
| S.H4 | HAH | Human | Han | high | 3750 | Daocheng | 30 | Male | c2 | 21 | 588 | 27484 |
| S.H9 | HAH | Human | Han | high | 3750 | Daocheng | 39 | Female | N/A | 21 | 141 | 28060 |
| S.H3 | HAH | Human | Han | high | 3750 | Daocheng | 40 | Female | N/A | 21 | 157 | 30212 |
| S.H48 | HAH | Human | Han | high | 3750 | Daocheng | 23 | Male | N/A | 21 | 769 | 30940 |
| S.H51 | HAH | Human | Han | high | 3750 | Daocheng | 23 | Male | c13 | 21 | 402 | 32138 |
| S.H20 | HAH | Human | Han | high | 3750 | Daocheng | 20 | Female | N/A | 21 | 1330 | 32495 |
| S.H37 | HAH | Human | Han | high | 3750 | Daocheng | 51 | Female | c9 | 21 | 501 | 32832 |
| S.H42 | HAH | Human | Han | high | 3750 | Daocheng | 34 | Female | N/A | 21 | 440 | 33797 |
| S.H21 | HAH | Human | Han | high | 3750 | Daocheng | 29 | Male | c6 | 21 | 312 | 39343 |
| S.H44 | HAH | Human | Han | high | 3750 | Daocheng | 48 | Female | c11 | 21 | 85 | 40151 |
| S.H53 | HAH | Human | Han | high | 3750 | Daocheng | 43 | Female | N/A | 21 | 1573 | 45640 |
| S.125 | HAT | Human | Tibetan | high | 3850 | Daocheng | 40 | Female | k33 | 22 | 48 | 3188 |
| S.62 | HAT | Human | Tibetan | high | 3850 | Daocheng | 13 | Male | m18 | 23 | 34 | 3878 |
| S.38 | HAT | Human | Tibetan | high | 3850 | Daocheng | 19 | Male | m12 | 23 | 39 | 4746 |
| S.126 | HAT | Human | Tibetan | high | 3850 | Daocheng | 15 | Male | k33 | 22 | 69 | 4933 |
| S.71 | HAT | Human | Tibetan | high | 3850 | Daocheng | 1 | Male | m20 | 23 | 46 | 5227 |
| S.44 | HAT | Human | Tibetan | high | 3850 | Daocheng | 5 | Male | m13 | 23 | 87 | 5361 |
| S.50 | HAT | Human | Tibetan | high | 3850 | Daocheng | 3 | Female | m15 | 23 | 127 | 5835 |
| S.29 | HAT | Human | Tibetan | high | 3850 | Daocheng | 23 | Female | m10 | 23 | 127 | 7154 |
| S.129 | HAT | Human | Tibetan | high | 3850 | Daocheng | 15 | Male | k34 | 22 | 38 | 7367 |
| S.40b | HAT | Human | Tibetan | high | 3850 | Daocheng | 76 | Male | m2 | 23 | 55 | 7786 |
| S.128 | HAT | Human | Tibetan | high | 3850 | Daocheng | 66 | Male | k34 | 22 | 77 | 8234 |
| S.78a | HAT | Human | Tibetan | high | 3850 | Daocheng | 19 | Female | m22 | 23 | 55 | 8300 |
| S.117 | HAT | Human | Tibetan | high | 3850 | Daocheng | 26 | Female | m31 | 23 | 41 | 8381 |
| S.46 | HAT | Human | Tibetan | high | 3850 | Daocheng | 43 | Male | m14 | 23 | 160 | 8810 |
| S.86 | HAT | Human | Tibetan | high | 3850 | Daocheng | 38 | Male | m24 | 23 | 109 | 8836 |
| S.49 | HAT | Human | Tibetan | high | 3850 | Daocheng | 26 | Female | m15 | 23 | 71 | 9020 |
| S.7 | HAT | Human | Tibetan | high | 3850 | Daocheng | 55 | Female | m3 | 23 | 291 | 9024 |
| S.74 | HAT | Human | Tibetan | high | 3850 | Daocheng | 3 | Female | m21 | 23 | 123 | 9033 |
| S.124 | HAT | Human | Tibetan | high | 3850 | Daocheng | 16 | Male | k32 | 22 | 47 | 9446 |
| S.15 | HAT | Human | Tibetan | high | 3850 | Daocheng | 40 | Male | m6 | 23 | 92 | 10479 |
| S114 | HAT | Human | Tibetan | high | 3850 | Daocheng | 16 | Male | m30 | 23 | 94 | 10986 |
| S.55 | HAT | Human | Tibetan | high | 3850 | Daocheng | 36 | Male | m16 | 23 | 93 | 11072 |
| S.135 | HAT | Human | Tibetan | high | 3850 | Daocheng | 4 | Male | k36 | 22 | 91 | 11503 |
| S.108 | HAT | Human | Tibetan | high | 3850 | Daocheng | 3 | Female | m29 | 23 | 120 | 11878 |
| S.30 | HAT | Human | Tibetan | high | 3850 | Daocheng | 15 | Male | m10 | 23 | 127 | 12192 |
| S.25 | HAT | Human | Tibetan | high | 3850 | Daocheng | 14 | Male | m9 | 23 | 56 | 12365 |
| S.42 | HAT | Human | Tibetan | high | 3850 | Daocheng | 16 | Female | m13 | 23 | 117 | 12433 |
| S.66 | HAT | Human | Tibetan | high | 3850 | Daocheng | 78 | Female | m19 | 23 | 177 | 12760 |
| S.105 | HAT | Human | Tibetan | high | 3850 | Daocheng | 22 | Male | m29 | 23 | 107 | 12805 |
| S.31 | HAT | Human | Tibetan | high | 3850 | Daocheng | 13 | Male | m10 | 23 | 97 | 12808 |
| S.133 | HAT | Human | Tibetan | high | 3850 | Daocheng | 72 | Female | k36 | 22 | 187 | 13187 |
| S.92 | HAT | Human | Tibetan | high | 3850 | Daocheng | 35 | Male | m26 | 23 | 80 | 13454 |
| S.2 | HAT | Human | Tibetan | high | 3850 | Daocheng | 23 | Male | m1 | 23 | 133 | 13814 |
| S.64 | HAT | Human | Tibetan | high | 3850 | Daocheng | 10 | Male | m18 | 23 | 129 | 13842 |
| S.22 | HAT | Human | Tibetan | high | 3850 | Daocheng | 66 | Female | m8 | 23 | 63 | 13879 |
| S.11 | HAT | Human | Tibetan | high | 3850 | Daocheng | 68 | Male | m5 | 23 | 61 | 13900 |
| S.70 | HAT | Human | Tibetan | high | 3850 | Daocheng | 52 | Male | m20 | 23 | 194 | 14066 |
| S19 | HAT | Human | Tibetan | high | 3850 | Daocheng | 63 | Female | m7 | 23 | 167 | 14121 |
| S.51 | HAT | Human | Tibetan | high | 3850 | Daocheng | 1 | Female | m15 | 23 | 164 | 14487 |

| | | | | | | | | | | | | |
|---|---|---|---|---|---|---|---|---|---|---|---|---|
| S.37 | HAT | Human | Tibetan | high | 3850 | Daocheng | 42 | Female | m12 | 23 | 262 | 14552 |
| S.24 | HAT | Human | Tibetan | high | 3850 | Daocheng | 61 | Female | m9 | 23 | 86 | 14740 |
| S.147 | HAT | Human | Tibetan | high | 3850 | Daocheng | 38 | Female | k41 | 22 | 225 | 14866 |
| S.142b | HAT | Human | Tibetan | high | 3850 | Daocheng | 13 | Female | k39 | 22 | 70 | 15655 |
| S.18 | HAT | Human | Tibetan | high | 3850 | Daocheng | 18 | Male | m6 | 23 | 98 | 15998 |
| S.40a | HAT | Human | Tibetan | high | 3850 | Daocheng | 76 | Male | m2 | 23 | 192 | 16764 |
| S.41 | HAT | Human | Tibetan | high | 3850 | Daocheng | 69 | Male | m13 | 23 | 199 | 16894 |
| S93 | HAT | Human | Tibetan | high | 3850 | Daocheng | 17 | Female | m26 | 23 | 90 | 17777 |
| S.35 | HAT | Human | Tibetan | high | 3850 | Daocheng | 11 | Male | m11 | 23 | 219 | 18295 |
| S.63 | HAT | Human | Tibetan | high | 3850 | Daocheng | 11 | Male | m18 | 23 | 237 | 18477 |
| S.8 | HAT | Human | Tibetan | high | 3850 | Daocheng | 57 | Female | m4 | 23 | 134 | 18823 |
| S.123 | HAT | Human | Tibetan | high | 3850 | Daocheng | 45 | Male | k32 | 22 | 45 | 19228 |
| S.81 | HAT | Human | Tibetan | high | 3850 | Daocheng | 47 | Male | m23 | 23 | 183 | 19353 |
| S.116 | HAT | Human | Tibetan | high | 3850 | Daocheng | 71 | Male | m31 | 23 | 113 | 19425 |
| S.43 | HAT | Human | Tibetan | high | 3850 | Daocheng | 11 | Female | m13 | 23 | 218 | 19676 |
| S103 | HAT | Human | Tibetan | high | 3850 | Daocheng | 66 | Male | m29 | 23 | 129 | 19798 |
| S.131 | HAT | Human | Tibetan | high | 3850 | Daocheng | 28 | Male | k35 | 22 | 62 | 19967 |
| S.57 | HAT | Human | Tibetan | high | 3850 | Daocheng | 63 | Female | m17 | 23 | 171 | 20069 |
| S.17 | HAT | Human | Tibetan | high | 3850 | Daocheng | 18 | Female | m6 | 23 | 244 | 20196 |
| S.79 | HAT | Human | Tibetan | high | 3850 | Daocheng | 6 | Female | m22 | 23 | 83 | 20395 |
| S.113 | HAT | Human | Tibetan | high | 3850 | Daocheng | 17 | Male | m30 | 23 | 229 | 20872 |
| S85 | HAT | Human | Tibetan | high | 3850 | Daocheng | 61 | Female | m24 | 23 | 220 | 21045 |
| S.102 | HAT | Human | Tibetan | high | 3850 | Daocheng | 12 | Female | m28 | 23 | 354 | 22125 |
| S.142a | HAT | Human | Tibetan | high | 3850 | Daocheng | 13 | Female | k39 | 22 | 60 | 22561 |
| S.33 | HAT | Human | Tibetan | high | 3850 | Daocheng | 58 | Female | m11 | 23 | 174 | 22692 |
| S.60 | HAT | Human | Tibetan | high | 3850 | Daocheng | 50 | Female | m18 | 23 | 76 | 23877 |
| S.72 | HAT | Human | Tibetan | high | 3850 | Daocheng | 57 | Male | m21 | 23 | 157 | 24369 |
| S.61 | HAT | Human | Tibetan | high | 3850 | Daocheng | 27 | Female | m18 | 23 | 87 | 24533 |
| S.9 | HAT | Human | Tibetan | high | 3850 | Daocheng | 12 | Male | m4 | 23 | 134 | 24589 |
| S.104 | HAT | Human | Tibetan | high | 3850 | Daocheng | 63 | Female | m29 | 23 | 117 | 24770 |
| S.78b | HAT | Human | Tibetan | high | 3850 | Daocheng | 19 | Female | m22 | 23 | 195 | 25200 |
| S.65 | HAT | Human | Tibetan | high | 3850 | Daocheng | 3 | Female | m18 | 23 | 122 | 26297 |
| S.73 | HAT | Human | Tibetan | high | 3850 | Daocheng | 12 | Female | m21 | 23 | 109 | 27050 |
| S.69 | HAT | Human | Tibetan | high | 3850 | Daocheng | 78 | Female | m20 | 23 | 238 | 27329 |
| S20 | HAT | Human | Tibetan | high | 3850 | Daocheng | 20 | Female | m7 | 23 | 116 | 27356 |
| S.28 | HAT | Human | Tibetan | high | 3850 | Daocheng | 68 | Female | m10 | 23 | 240 | 28711 |
| S.107 | HAT | Human | Tibetan | high | 3850 | Daocheng | 4 | Female | m29 | 23 | 170 | 28945 |
| S.138 | HAT | Human | Tibetan | high | 3850 | Daocheng | 17 | Male | k38 | 22 | 350 | 30577 |
| S.1 | HAT | Human | Tibetan | high | 3850 | Daocheng | 45 | Male | m1 | 23 | 103 | 31490 |
| S96 | HAT | Human | Tibetan | high | 3850 | Daocheng | 37 | Male | m27 | 23 | 130 | 31550 |
| S.54 | HAT | Human | Tibetan | high | 3850 | Daocheng | 66 | Male | m16 | 23 | 298 | 32088 |
| S.77 | HAT | Human | Tibetan | high | 3850 | Daocheng | 47 | Female | m22 | 23 | 375 | 32184 |
| S.112 | HAT | Human | Tibetan | high | 3850 | Daocheng | 66 | Male | m30 | 23 | 326 | 32202 |
| S.98 | HAT | Human | Tibetan | high | 3850 | Daocheng | 12 | Female | m27 | 23 | 334 | 32243 |
| S3 | HAT | Human | Tibetan | high | 3850 | Daocheng | 4 | Male | m1 | 23 | 160 | 34478 |
| S.80 | HAT | Human | Tibetan | high | 3850 | Daocheng | 3 | Female | m22 | 23 | 85 | 36831 |
| S.118 | HAT | Human | Tibetan | high | 3850 | Daocheng | 18 | Male | m31 | 23 | 192 | 37467 |
| S.111 | HAT | Human | Tibetan | high | 3850 | Daocheng | 65 | Female | m30 | 23 | 167 | 37777 |
| S.5 | HAT | Human | Tibetan | high | 3850 | Daocheng | 47 | Female | m2 | 23 | 128 | 38911 |
| S.76 | HAT | Human | Tibetan | high | 3850 | Daocheng | 81 | Female | m22 | 23 | 298 | 39601 |
| S.106 | HAT | Human | Tibetan | high | 3850 | Daocheng | 29 | Female | m29 | 23 | 167 | 39729 |
| S.58 | HAT | Human | Tibetan | high | 3850 | Daocheng | 19 | Female | m17 | 23 | 294 | 44289 |

| | | | | | | | | | | | | |
|---|---|---|---|---|---|---|---|---|---|---|---|---|
| S.83 | HAT | Human | Tibetan | high | 3850 | Daocheng | 1 | Female | m23 | 23 | 384 | 44446 |
| S.97 | HAT | Human | Tibetan | high | 3850 | Daocheng | 15 | Male | m27 | 23 | 242 | 44975 |
| S.145 | HAT | Human | Tibetan | high | 3850 | Daocheng | 46 | Female | k40 | 22 | 214 | 44981 |
| S.134 | HAT | Human | Tibetan | high | 3850 | Daocheng | 13 | Female | k36 | 22 | 190 | 47530 |
| S.67 | HAT | Human | Tibetan | high | 3850 | Daocheng | 62 | Female | m19 | 23 | 245 | 47943 |
| S88 | HAT | Human | Tibetan | high | 3850 | Daocheng | 58 | Female | m25 | 23 | 256 | 49945 |
| S100 | HAT | Human | Tibetan | high | 3850 | Daocheng | 70 | Male | m28 | 23 | 130 | 50427 |
| S119 | HAT | Human | Tibetan | high | 3850 | Daocheng | 3 | Male | m31 | 23 | 298 | 59444 |
| S16 | HAT | Human | Tibetan | high | 3850 | Daocheng | 48 | Female | m6 | 23 | 192 | 70407 |
| S34 | HAT | Human | Tibetan | high | 3850 | Daocheng | 12 | Male | m11 | 23 | 361 | 113650 |
| S.14b | HATP | Pig | Tibetan_pig | high | 3850 | Daocheng | >1 | N/A | m5 | 23 | 57 | 5659 |
| S.39b | HATP | Pig | Tibetan_pig | high | 3850 | Daocheng | >1 | N/A | m12 | 23 | 163 | 7192 |
| S.127b | HATP | Pig | Tibetan_pig | high | 3850 | Daocheng | >1 | N/A | k34 | 22 | 55 | 7738 |
| S.27b | HATP | Pig | Tibetan_pig | high | 3850 | Daocheng | >1 | N/A | m9 | 23 | 81 | 9546 |
| S.130b | HATP | Pig | Tibetan_pig | high | 3850 | Daocheng | >1 | N/A | k35 | 22 | 147 | 9589 |
| S.132e | HATP | Pig | Tibetan_pig | high | 3850 | Daocheng | >1 | N/A | k36 | 22 | 165 | 10382 |
| S.132a | HATP | Pig | Tibetan_pig | high | 3850 | Daocheng | >1 | N/A | k36 | 22 | 150 | 12462 |
| S.122 | HATP | Pig | Tibetan_pig | high | 3850 | Daocheng | >1 | N/A | k33 | 22 | 209 | 13976 |
| S.10a | HATP | Pig | Tibetan_pig | high | 3850 | Daocheng | >1 | N/A | m4 | 23 | 232 | 14296 |
| S.127a | HATP | Pig | Tibetan_pig | high | 3850 | Daocheng | >1 | N/A | k34 | 22 | 117 | 15044 |
| S.27a | HATP | Pig | Tibetan_pig | high | 3850 | Daocheng | >1 | N/A | m9 | 23 | 198 | 15099 |
| S.121a | HATP | Pig | Tibetan_pig | high | 3850 | Daocheng | >1 | N/A | k32 | 22 | 267 | 15362 |
| S.121b | HATP | Pig | Tibetan_pig | high | 3850 | Daocheng | >1 | N/A | k32 | 22 | 208 | 16411 |
| S.59 | HATP | Pig | Tibetan_pig | high | 3850 | Daocheng | >1 | N/A | m17 | 23 | 170 | 17995 |
| S.14a | HATP | Pig | Tibetan_pig | high | 3850 | Daocheng | >1 | N/A | m5 | 23 | 108 | 18411 |
| S.146c | HATP | Pig | Tibetan_pig | high | 3850 | Daocheng | >1 | N/A | k41 | 22 | 230 | 20115 |
| S.146a | HATP | Pig | Tibetan_pig | high | 3850 | Daocheng | >1 | N/A | k41 | 22 | 125 | 21343 |
| S39a | HATP | Pig | Tibetan_pig | high | 3850 | Daocheng | >1 | N/A | m12 | 23 | 126 | 22862 |
| S.132b | HATP | Pig | Tibetan_pig | high | 3850 | Daocheng | >1 | N/A | k36 | 22 | 195 | 28574 |
| S.132c | HATP | Pig | Tibetan_pig | high | 3850 | Daocheng | >1 | N/A | k36 | 22 | 168 | 28616 |
| S.68 | HATP | Pig | Tibetan_pig | high | 3850 | Daocheng | >1 | N/A | m19 | 23 | 246 | 29602 |
| S.146b | HATP | Pig | Tibetan_pig | high | 3850 | Daocheng | >1 | N/A | k41 | 22 | 277 | 44333 |
| S.132d | HATP | Pig | Tibetan_pig | high | 3850 | Daocheng | >1 | N/A | k36 | 22 | 251 | 45603 |
| YA39 | LAH | Human | Han | low | 641 | Yaan | 22 | Male | N/A | 26 | 370 | 3816 |
| YA33 | LAH | Human | Han | low | 641 | Yaan | 23 | Male | N/A | 26 | 314 | 4022 |
| YA36 | LAH | Human | Han | low | 641 | Yaan | 23 | Male | N/A | 26 | 573 | 5451 |
| YA25 | LAH | Human | Han | low | 641 | Yaan | 21 | Female | N/A | 26 | 564 | 5469 |
| YA14 | LAH | Human | Han | low | 641 | Yaan | 22 | Male | N/A | 26 | 410 | 5935 |
| YA22 | LAH | Human | Han | low | 641 | Yaan | 21 | Male | N/A | 26 | 424 | 6125 |
| WJ3 | LAH | Human | Han | low | 515 | Chengdu | 29 | Male | N/A | 16 | 315 | 6717 |
| YA17 | LAH | Human | Han | low | 641 | Yaan | 23 | Male | N/A | 26 | 390 | 6732 |
| YA26 | LAH | Human | Han | low | 641 | Yaan | 21 | Female | N/A | 26 | 559 | 7218 |
| YA8 | LAH | Human | Han | low | 641 | Yaan | 21 | Male | N/A | 26 | 539 | 7422 |
| YA34 | LAH | Human | Han | low | 641 | Yaan | 22 | Male | N/A | 26 | 427 | 8174 |
| WJ4 | LAH | Human | Han | low | 515 | Chengdu | N/A | N/A | N/A | 16 | 380 | 8406 |
| YA19 | LAH | Human | Han | low | 641 | Yaan | 19 | Female | N/A | 26 | 593 | 8575 |
| YA35 | LAH | Human | Han | low | 641 | Yaan | 22 | Male | N/A | 26 | 407 | 8586 |
| YA23 | LAH | Human | Han | low | 641 | Yaan | 21 | Male | N/A | 26 | 531 | 8858 |
| YA18 | LAH | Human | Han | low | 641 | Yaan | 21 | Female | N/A | 26 | 529 | 9196 |
| YA28 | LAH | Human | Han | low | 641 | Yaan | 20 | Female | N/A | 26 | 408 | 9404 |
| YA32 | LAH | Human | Han | low | 641 | Yaan | 22 | Male | N/A | 26 | 494 | 9817 |
| WJ12 | LAH | Human | Han | low | 515 | Chengdu | 56 | Male | N/A | 16 | 344 | 10082 |

| | | | | | | | | | | | | |
|---|---|---|---|---|---|---|---|---|---|---|---|---|
| YA11 | LAH | Human | Han | low | 641 | Yaan | 21 | Male | N/A | 26 | 496 | 10883 |
| WJ9 | LAH | Human | Han | low | 515 | Chengdu | 25 | Male | N/A | 16 | 395 | 10960 |
| WJ15 | LAH | Human | Han | low | 515 | Chengdu | 23 | Female | N/A | 16 | 408 | 12530 |
| YA37 | LAH | Human | Han | low | 641 | Yaan | 23 | Male | N/A | 26 | 497 | 12655 |
| YA29 | LAH | Human | Han | low | 641 | Yaan | 22 | Female | N/A | 26 | 689 | 12895 |
| YA27 | LAH | Human | Han | low | 641 | Yaan | 20 | Female | N/A | 26 | 636 | 13005 |
| YA31 | LAH | Human | Han | low | 641 | Yaan | 22 | Male | N/A | 26 | 539 | 13030 |
| YA24 | LAH | Human | Han | low | 641 | Yaan | 23 | Male | N/A | 26 | 392 | 13112 |
| YA38 | LAH | Human | Han | low | 641 | Yaan | 21 | Male | N/A | 26 | 406 | 13189 |
| YA15 | LAH | Human | Han | low | 641 | Yaan | 22 | Male | N/A | 26 | 891 | 13568 |
| YA1 | LAH | Human | Han | low | 641 | Yaan | 21 | Male | N/A | 26 | 568 | 13857 |
| YA7 | LAH | Human | Han | low | 641 | Yaan | 21 | Male | N/A | 26 | 554 | 14114 |
| YA5 | LAH | Human | Han | low | 641 | Yaan | 20 | Male | N/A | 26 | 759 | 14716 |
| YA30 | LAH | Human | Han | low | 641 | Yaan | 22 | Male | N/A | 26 | 493 | 14895 |
| WJ5 | LAH | Human | Han | low | 515 | Chengdu | 23 | Male | N/A | 16 | 385 | 15268 |
| YA9 | LAH | Human | Han | low | 641 | Yaan | 21 | Male | N/A | 26 | 502 | 15809 |
| YA6 | LAH | Human | Han | low | 641 | Yaan | 21 | Male | N/A | 26 | 478 | 16134 |
| YA20 | LAH | Human | Han | low | 641 | Yaan | 22 | Female | N/A | 26 | 752 | 16243 |
| WJ7 | LAH | Human | Han | low | 515 | Chengdu | 25 | Male | N/A | 16 | 596 | 16337 |
| WJ13 | LAH | Human | Han | low | 515 | Chengdu | 30 | Female | N/A | 16 | 502 | 16930 |
| YA3 | LAH | Human | Han | low | 641 | Yaan | 21 | Male | N/A | 26 | 621 | 17510 |
| YA13 | LAH | Human | Han | low | 641 | Yaan | 22 | Male | N/A | 26 | 957 | 18295 |
| YA10 | LAH | Human | Han | low | 641 | Yaan | 22 | Male | N/A | 26 | 421 | 19381 |
| WJ14 | LAH | Human | Han | low | 515 | Chengdu | 29 | Female | N/A | 16 | 539 | 20063 |
| YA12 | LAH | Human | Han | low | 641 | Yaan | 24 | Male | N/A | 26 | 848 | 20645 |
| YA16 | LAH | Human | Han | low | 641 | Yaan | 21 | Male | N/A | 26 | 812 | 23052 |
| YA4 | LAH | Human | Han | low | 641 | Yaan | 22 | Male | N/A | 26 | 924 | 23965 |
| WJ11 | LAH | Human | Han | low | 515 | Chengdu | 27 | Male | N/A | 16 | 545 | 24355 |
| YA2 | LAH | Human | Han | low | 641 | Yaan | 22 | Male | N/A | 26 | 615 | 24422 |
| LQS4 | LAQP | Pig | Qingyu_pig | low | 365 | Bazhong | >1 | N/A | N/A | 25 | 567 | 6166 |
| LQS12 | LAQP | Pig | Qingyu_pig | low | 510 | Bazhong | >1 | boar | N/A | 29 | 857 | 10036 |
| LQS14 | LAQP | Pig | Qingyu_pig | low | 510 | Bazhong | >1 | boar | N/A | 29 | 1691 | 10795 |
| LQS10 | LAQP | Pig | Qingyu_pig | low | 365 | Bazhong | 0.5 | N/A | N/A | 25 | 979 | 11687 |
| LQS9 | LAQP | Pig | Qingyu_pig | low | 365 | Bazhong | 0.5 | N/A | N/A | 25 | 771 | 14086 |
| LQS18 | LAQP | Pig | Qingyu_pig | low | 510 | Bazhong | >1 | boar | N/A | 29 | 1596 | 14265 |
| LQS5 | LAQP | Pig | Qingyu_pig | low | 365 | Bazhong | >1 | N/A | N/A | 25 | 878 | 15746 |
| LQS2 | LAQP | Pig | Qingyu_pig | low | 365 | Bazhong | >1 | N/A | N/A | 25 | 882 | 18987 |
| LQS3 | LAQP | Pig | Qingyu_pig | low | 365 | Bazhong | >1 | N/A | N/A | 25 | 1345 | 19585 |
| LQS6 | LAQP | Pig | Qingyu_pig | low | 365 | Bazhong | 0.5 | N/A | N/A | 25 | 977 | 19728 |
| LQS11 | LAQP | Pig | Qingyu_pig | low | 365 | Bazhong | 0.5 | N/A | N/A | 25 | 1672 | 20902 |
| LQS8 | LAQP | Pig | Qingyu_pig | low | 365 | Bazhong | 0.5 | N/A | N/A | 25 | 1535 | 21326 |
| LQS1 | LAQP | Pig | Qingyu_pig | low | 365 | Bazhong | >1 | N/A | N/A | 25 | 1203 | 21746 |
| LQS17 | LAQP | Pig | Qingyu_pig | low | 510 | Bazhong | >1 | boar | N/A | 29 | 1325 | 25223 |
| LQS13 | LAQP | Pig | Qingyu_pig | low | 510 | Bazhong | >1 | boar | N/A | 29 | 2560 | 33046 |
| LRS.M5 | LARP | Pig | Rongchang_pig | low | 319 | Chongqing | 0.5 | sow | N/A | 22.7 | 792 | 3804 |
| LRS12 | LARP | Pig | Rongchang_pig | low | 319 | Chongqing | 0.5 | boar | N/A | 22.7 | 545 | 4123 |
| LRS.M2 | LARP | Pig | Rongchang_pig | low | 319 | Chongqing | 0.5 | sow | N/A | 22.7 | 873 | 9949 |
| LRS6 | LARP | Pig | Rongchang_pig | low | 319 | Chongqing | 0.5 | boar | N/A | 22.7 | 1500 | 13344 |
| LRS1 | LARP | Pig | Rongchang_pig | low | 319 | Chongqing | >1 | boar | N/A | 22.7 | 1428 | 13533 |
| LRS14 | LARP | Pig | Rongchang_pig | low | 319 | Chongqing | 0.5 | boar | N/A | 22.7 | 1484 | 14369 |
| LRS.M10 | LARP | Pig | Rongchang_pig | low | 319 | Chongqing | 0.5 | sow | N/A | 22.7 | 1298 | 15203 |
| LRS.M12 | LARP | Pig | Rongchang_pig | low | 319 | Chongqing | 0.5 | sow | N/A | 22.7 | 1782 | 15663 |

| | | | | | | | | | | | | |
|---|---|---|---|---|---|---|---|---|---|---|---|---|
| LRS.M4 | LARP | Pig | Rongchang_pig | low | 319 | Chongqing | 0.5 | sow | N/A | 22.7 | 1794 | 18151 |
| LRS.M11 | LARP | Pig | Rongchang_pig | low | 319 | Chongqing | 0.5 | sow | N/A | 22.7 | 1664 | 18316 |
| LRS18 | LARP | Pig | Rongchang_pig | low | 319 | Chongqing | 0.5 | boar | N/A | 22.7 | 1702 | 18879 |
| LRS.M3 | LARP | Pig | Rongchang_pig | low | 319 | Chongqing | 0.5 | sow | N/A | 22.7 | 2210 | 19602 |
| LRS3 | LARP | Pig | Rongchang_pig | low | 319 | Chongqing | >1 | boar | N/A | 22.7 | 1762 | 20307 |
| LRS9 | LARP | Pig | Rongchang_pig | low | 319 | Chongqing | 0.5 | boar | N/A | 22.7 | 1974 | 21868 |
| LRS7 | LARP | Pig | Rongchang_pig | low | 319 | Chongqing | 0.5 | boar | N/A | 22.7 | 2270 | 23223 |
| LRS16 | LARP | Pig | Rongchang_pig | low | 319 | Chongqing | 0.5 | boar | N/A | 22.7 | 2172 | 28630 |
| LRS.M8 | LARP | Pig | Rongchang_pig | low | 319 | Chongqing | 0.5 | sow | N/A | 22.7 | 2585 | 28759 |
| LRS8 | LARP | Pig | Rongchang_pig | low | 319 | Chongqing | 0.5 | boar | N/A | 22.7 | 2207 | 30538 |
| LRS2 | LARP | Pig | Rongchang_pig | low | 319 | Chongqing | 0.5 | boar | N/A | 22.7 | 2595 | 31625 |
| LRS5 | LARP | Pig | Rongchang_pig | low | 319 | Chongqing | 0.5 | boar | N/A | 22.7 | 3345 | 41728 |
| LTS5 | LATP | Pig | Tibetan_pig | low | 454 | Jianyang | >1 | boar | N/A | 20 | 381 | 4471 |
| LTS16 | LATP | Pig | Tibetan_pig | low | 454 | Jianyang | >1 | boar | N/A | 20 | 623 | 6928 |
| LTS13 | LATP | Pig | Tibetan_pig | low | 454 | Jianyang | >1 | boar | N/A | 20 | 901 | 11472 |
| LTS8 | LATP | Pig | Tibetan_pig | low | 454 | Jianyang | >1 | boar | N/A | 20 | 799 | 11561 |
| LTS4 | LATP | Pig | Tibetan_pig | low | 454 | Jianyang | >1 | boar | N/A | 20 | 1276 | 14336 |
| LTS1 | LATP | Pig | Tibetan_pig | low | 454 | Jianyang | >1 | boar | N/A | 20 | 1014 | 14830 |
| LTS17 | LATP | Pig | Tibetan_pig | low | 454 | Jianyang | >1 | boar | N/A | 20 | 1365 | 15134 |
| S.K4 | LATP | Pig | Tibetan_pig | low | 1430 | Kangding | >1 | N/A | N/A | 21 | 1541 | 15828 |
| LTS11 | LATP | Pig | Tibetan_pig | low | 454 | Jianyang | >1 | boar | N/A | 20 | 596 | 16710 |
| S.K7 | LATP | Pig | Tibetan_pig | low | 1430 | Kangding | >1 | N/A | N/A | 21 | 1955 | 20556 |
| LTS6 | LATP | Pig | Tibetan_pig | low | 454 | Jianyang | >1 | boar | N/A | 20 | 554 | 21266 |
| LTS7 | LATP | Pig | Tibetan_pig | low | 454 | Jianyang | >1 | boar | N/A | 20 | 1348 | 21892 |
| LTS18 | LATP | Pig | Tibetan_pig | low | 454 | Jianyang | >1 | boar | N/A | 20 | 791 | 21956 |
| LTS3 | LATP | Pig | Tibetan_pig | low | 454 | Jianyang | >1 | boar | N/A | 20 | 1154 | 25169 |
| LTS15 | LATP | Pig | Tibetan_pig | low | 454 | Jianyang | >1 | boar | N/A | 20 | 734 | 27590 |
| S.K3 | LATP | Pig | Tibetan_pig | low | 1430 | Kangding | >1 | N/A | N/A | 21 | 1899 | 28204 |
| LTS10 | LATP | Pig | Tibetan_pig | low | 454 | Jianyang | >1 | boar | N/A | 20 | 1049 | 31362 |
| LTS9 | LATP | Pig | Tibetan_pig | low | 454 | Jianyang | >1 | boar | N/A | 20 | 1450 | 31926 |
| LTS12 | LATP | Pig | Tibetan_pig | low | 454 | Jianyang | >1 | boar | N/A | 20 | 1908 | 32988 |
| LTS2 | LATP | Pig | Tibetan_pig | low | 454 | Jianyang | >1 | boar | N/A | 20 | 1101 | 36746 |
| S.K6 | LATP | Pig | Tibetan_pig | low | 1430 | Kangding | >1 | N/A | N/A | 21 | 2484 | 37691 |
| S.K8 | LATP | Pig | Tibetan_pig | low | 1430 | Kangding | >1 | N/A | N/A | 21 | 1555 | 40947 |
| LTS14 | LATP | Pig | Tibetan_pig | low | 454 | Jianyang | >1 | boar | N/A | 20 | 1186 | 44532 |
| S.K5 | LATP | Pig | Tibetan_pig | low | 1430 | Kangding | >1 | N/A | N/A | 21 | 2935 | 61338 |

Table S2: PERMANOVA pseudo-F and p-values associated with specific demographic factor-microbiome associations.

| | Factors | Variables (Groups) | Unweighted UniFrac | | Bray–Curtis | |
|---|---|---|---|---|---|---|
| | | | **pseudo-F** | **p-value** | **pseudo-F** | **p-value** |
| Human | Altitude | 2 (high/low) | 28.608 | 0.001 | 34.165 | 0.001 |
| | Elevation | 5 | 10.083 | 0.001 | 11.394 | 0.001 |
| | Race | 2 (Han/Tibetan) | 23.954 | 0.001 | 21.982 | 0.001 |
| | Age (years) | 66 (0.5−81 years) | 1.425 | 0.001 | 1.518 | 0.001 |
| | Gender | 3 (male/female/unclear) | 1.160 | 0.125 | 1.439 | 0.047 |
| | Family | 54 | 1.711 | 0.001 | 1.883 | 0.001 |
| | Height | 41 | 1.155 | 0.003 | 1.200 | 0.003 |
| | Weight | 35 | 1.595 | 0.001 | 1.582 | 0.001 |
| | Temperature | 5 (16−26℃) | 10.083 | 0.001 | 11.394 | 0.001 |
| Pig | Altitude | 2 (high/low) | 22.421 | 0.001 | 19.365 | 0.001 |
| | Elevation | 7 | 7.304 | 0.001 | 13.639 | 0.001 |
| | Breed | 3 | 6.112 | 0.001 | 11.948 | 0.001 |
| | Gender | 3 (boar/saw/unclear) | 6.464 | 0.001 | 8.516 | 0.001 |
| | Family | 13 | 3.036 | 0.001 | 3.436 | 0.001 |
| | Temperature | 7 (20～29℃) | 7.304 | 0.001 | 13.639 | 0.001 |
| | Location | 6 | 8.338 | 0.001 | 15.643 | 0.001 |

*number of permutations: 999

Table S3: Core microbiome identified from high-altitude groups.

| #OTU_ID | #Taxonomy |
|---|---|
| denovo99917 | p__Actinobacteria, c__Actinobacteria, o__Actinomycetales, f__Cellulomonadaceae |
| denovo109171 | p__Actinobacteria, c__Actinobacteria, o__Actinomycetales, f__Dermabacteraceae, g__Brachybacterium, s__ |
| denovo58246 | p__Actinobacteria, c__Actinobacteria, o__Actinomycetales, f__Micrococcaceae, g__, s__ |
| denovo15788 | p__Actinobacteria, c__Actinobacteria, o__Actinomycetales, f__Micrococcaceae, g__Arthrobacter, s__ |
| denovo67645 | p__Actinobacteria, c__Actinobacteria, o__Actinomycetales, f__Micrococcaceae, g__Arthrobacter, s__ |
| denovo51778 | p__Actinobacteria, c__Actinobacteria, o__Actinomycetales, f__Micrococcaceae, g__Kocuria, s__palustris |
| denovo35373 | p__Cyanobacteria, c__Chloroplast, o__Streptophyta, f__, g__, s__ |
| denovo110307 | p__Firmicutes, c__Bacilli, o__Bacillales |
| denovo8729 | p__Firmicutes, c__Bacilli, o__Bacillales, f__Paenibacillaceae, g__Paenibacillus, s__ |
| denovo74318 | p__Firmicutes, c__Bacilli, o__Bacillales, f__Planococcaceae, g__, s__ |
| denovo13766 | p__Firmicutes, c__Bacilli, o__Bacillales, f__Planococcaceae, g__Planomicrobium, s__ |
| denovo62900 | p__Firmicutes, c__Bacilli, o__Lactobacillales, f__Aerococcaceae, g__Aerococcus, s__ |
| denovo102650 | p__Firmicutes, c__Bacilli, o__Lactobacillales, f__Carnobacteriaceae, g__Carnobacterium, s__ |
| denovo66332 | p__Firmicutes, c__Bacilli, o__Lactobacillales, f__Enterococcaceae, g__Enterococcus, s__ |
| denovo750 | p__Firmicutes, c__Bacilli, o__Lactobacillales, f__Streptococcaceae, g__Lactococcus, s__ |

| | |
|---|---|
| denovo10529 | p__Firmicutes, c__Bacilli, o__Turicibacterales, f__Turicibacteraceae, g__Turicibacter, s__ |
| denovo45366 | p__Firmicutes, c__Clostridia, o__Clostridiales, f__Clostridiaceae, g__, s__ |
| denovo4749 | p__Firmicutes, c__Clostridia, o__Clostridiales, f__Ruminococcaceae, g__, s__ |
| denovo101460 | p__Proteobacteria, c__Alphaproteobacteria, o__Rhizobiales, f__Brucellaceae, g__Ochrobactrum, s__ |
| denovo23993 | p__Proteobacteria, c__Alphaproteobacteria, o__Sphingomonadales, f__Sphingomonadaceae, g__Sphingomonas, s__ |
| denovo78559 | p__Proteobacteria, c__Betaproteobacteria, o__Burkholderiales, f__Burkholderiaceae, g__Burkholderia, s__bryophila |
| denovo18534 | p__Proteobacteria, c__Gammaproteobacteria, o__Enterobacteriales, f__Enterobacteriaceae, g__, s__ |
| denovo113525 | p__Proteobacteria, c__Gammaproteobacteria, o__Enterobacteriales, f__Enterobacteriaceae, g__, s__ |
| denovo76706 | p__Proteobacteria, c__Gammaproteobacteria, o__Enterobacteriales, f__Enterobacteriaceae, g__Erwinia, s__ |
| denovo90479 | p__Proteobacteria, c__Gammaproteobacteria, o__Pseudomonadales, f__Moraxellaceae, g__, s__ |
| denovo32758 | p__Proteobacteria, c__Gammaproteobacteria, o__Pseudomonadales, f__Moraxellaceae, g__Acinetobacter, s__ |
| denovo76229 | p__Proteobacteria, c__Gammaproteobacteria, o__Pseudomonadales, f__Moraxellaceae, g__Acinetobacter, s__ |
| denovo1059 | p__Proteobacteria, c__Gammaproteobacteria, o__Pseudomonadales, f__Moraxellaceae, g__Enhydrobacter, s__ |
| denovo74768 | p__Proteobacteria, c__Gammaproteobacteria, o__Pseudomonadales, f__Moraxellaceae, g__Psychrobacter, s__sanguinis |
| denovo15170 | p__Proteobacteria, c__Gammaproteobacteria, o__Pseudomonadales, f__Pseudomonadaceae, g__Pseudomonas, s__ |
| denovo103576 | p__Proteobacteria, c__Gammaproteobacteria, o__Pseudomonadales, f__Pseudomonadaceae, g__Pseudomonas, s__ |
| denovo59828 | p__Proteobacteria, c__Gammaproteobacteria, o__Pseudomonadales, f__Pseudomonadaceae, g__Pseudomonas, s__viridiflava |
| denovo99305 | p__Proteobacteria, c__Gammaproteobacteria, o__Xanthomonadales, f__Xanthomonadaceae, g__, s__ |

#Core OTUs across 50 % of samples

## 2. Commandlines used for suquence analysis

**#Usearch commandlines for FASTQ** (Use one sample LQS1 as example)

#Active the Usearch file to make it executable in your Linux system

```
chmod +x /home/qiime/Desktop/Shared_Folder/raw_test/usearch9
```

#Check FASTQ files

```
./usearch9 -fastq_chars LQS1_1.fq -log chars.log
```

#Merge paired FASTQ files (Overlap)

```
./usearch9 -fastq_mergepairs LQS1_1.fq -reverse LQS1_2.fq -fastqout LQS1.fastq -fastq_minovlen 20 -fastq_maxdiffs 10
```

# Quality filter FASTQ and convert reads to QIIME FASTA format

```
./usearch9 -fastq_filter LQS1.fastq -fastaout LQS1.fasta -fastq_minlen 200 -fastq_maxee 0.5 -relabel LQS1_
```

**#Pyhton commandline in QIIME**

#Merge clean fasta files of all samples

```
cat LQS1.fasta LRS.M2.fasta LRS.M3.fasta ....... S.1.fasta S.2.fasta S.5.fasta S.7.fasta S.8.fasta S.9.fasta ->all.fasta
```

**# *de novo* OTU picking**

```
pick_de_novo_otus.py -i all.fasta -o all_denovo_otus/ -a -O 6
```

**#Quality control of OTU metadata**

#Discard singleton OTUs (OTU reads <2) from OTU table (biom file)

```
filter_otus_from_otu_table.py -i otu_table.biom -o otu_table_n2.biom -n 2
```

#Discard singleton OTUs sequences from OTU representative fasta files

```
filter_fasta.py -f all_rep_set.fasta -o all_rep_set_n2.fasta -b otu_table_n2.biom
```

# Chimera detection using Usearch again

```
./usearch9 -uchime_ref all_rep_set_n2.fasta -db silva.gold.ng.fasta -nonchimeras
all_rep_set_n2_nochim.fasta -chimeras chimera.fasta -strand plus
```

#Filter the chimera data from biom and tree files

```
filter_otus_from_otu_table.py -i otu_table_n2.biom -o otu_table_n2_nochim.biom -e chimera.fasta
filter_tree.py -i rep_set.tre -f all_rep_set_n2_nochim.fasta -o rep_set_n2_nochim.tre
```

# summarize and check the information of the final clean OTU table

```
biom summarize-table -i otu_table_n2_nochim.biom -o otu_sum.txt
biom summarize-table -i otu_table_n2_nochim.biom -o otu_sum_q.txt --qualitative
```

# Checks sample metadata mapping file (valid format) before diversity analysis

```
validate_mapping_file.py -m map_skin_all.txt -o sample_map_output/ -p -b
```

**#Alpha diversity analysis**

```
echo "alpha_diversity:metrics
shannon,chao1,observed_species,goods_coverage,simpson_reciprocal" > alpha_params.txt
alpha_rarefaction.py -i otu_table_n2_nochim.biom -m map_skin_all.txt -o a_div_n30 -p
alpha_params.txt -t rep_set_n2_nochim.tre -n 30 -a -O 4
```

**#Beta diversity analysis**

#Unweighted and weighted Unifrac

```
jackknifed_beta_diversity.py -i otu_table_n2_nochim.biom -m map_skin_all.txt -o jackknifed_b_div/
-t rep_set_n2_nochim.tre -e 3188
```

#Bray_curtis

```
beta_diversity.py -i jackknifed_b_div//rarefaction/ -o jackknifed_b_div//bray_curtis//rare_dm/ -m
bray_curtis
principal_coordinates.py -i jackknifed_b_div//bray_curtis//rare_dm/ -o
jackknifed_b_div//bray_curtis//pcoa/
make_emperor.py -i jackknifed_b_div//bray_curtis//pcoa/ -o
jackknifed_b_div//bray_curtis//emperor_pcoa_plots/ -m map_skin_all.txt
```

# Obtain the paired group distance data from (e.g. unweighted unifrac ) distance matrix

```
make_distance_boxplots.py -m map_skin_all.txt -d unweighted_unifrac_rarefaction_3188.txt -f
group -o group_sig_unweighted --save_raw_data
```

**#UPGMA tree**

```
upgma_cluster.py -i unweighted_unifrac_rarefaction_3188.txt -o unweighted_unifrac_UPGMA_tree
```

**#Taxa structure analysis**

```
summarize_taxa.py -i otu_table_n2_nochim.biom -o taxa
```

#plot bar chart using L6 or L2 level (genus or phylum) taxa abundance data

```
plot_taxa_summary.py -i a_L6.txt -c bar -o a/ -m -s -d 1200 -x 200 -y 10 -t svg
```

**#Core microbiome analysis**

#Filters samples from an OTU table

```
filter_samples_from_otu_table.py -i otu_table_n2_nochim.biom -o otu_table_HA.biom --
sample_id_fp list_HA.txt
```

#Identify the core microbiome

```
compute_core_microbiome.py -i otu_table_HA.biom -o otu_table_core_HA
```
